# Dissection of Insertion–Deletion Variants within Differentially Expressed Genes Involved in Wood Formation in *Populus*

**DOI:** 10.3389/fpls.2017.02199

**Published:** 2018-01-18

**Authors:** Chenrui Gong, Qingzhang Du, Jianbo Xie, Mingyang Quan, Beibei Chen, Deqiang Zhang

**Affiliations:** ^1^National Engineering Laboratory for Tree Breeding, College of Biological Sciences and Technology, Beijing Forestry University, Beijing, China; ^2^Key Laboratory of Genetics and Breeding in Forest Trees and Ornamental Plants, Ministry of Education, College of Biological Sciences and Technology, Beijing Forestry University, Beijing, China; ^3^College of Forestry, Henan Agricultural University, Zhengzhou, China; ^4^Beijing Advanced Innovation Center for Tree Breeding by Molecular Design, Beijing Forestry University, Beijing, China

**Keywords:** association genetics, epistasis networks, high-throughput sequencing, InDel variants, wood formation

## Abstract

Short insertions and deletions (InDels) are one of the major genetic variants and are distributed widely across the genome; however, few investigations of InDels have been conducted in long-lived perennial plants. Here, we employed a combination of RNA-seq and population resequencing to identify InDels within differentially expressed (DE) genes underlying wood formation in a natural population of *Populus tomentosa* (435 individuals) and utilized InDel-based association mapping to detect the causal variants under additive, dominance, and epistasis underlying growth and wood properties. In the present paper, 5,482 InDels detected from 629 DE genes showed uneven distributions throughout all 19 chromosomes, and 95.9% of these loci were diallelic InDels. Seventy-four InDels (positive false discovery rate *q* ≤ 0.10) from 68 genes exhibited significant additive/dominant effects on 10 growth and wood-properties, with an average of 14.7% phenotypic variance explained. Potential pleiotropy was observed in one-third of the InDels (representing 24 genes). Seven genes exhibited significantly differential expression among the genotypic classes of associated InDels, indicating possible important roles for these InDels. Epistasis analysis showed that overlapping interacting genes formed unique interconnected networks for each trait, supporting the putative biochemical links that control quantitative traits. Therefore, the identification and utilization of InDels in trees will be recognized as an effective marker system for molecular marker-assisted breeding applications, and further facilitate our understanding of quantitative genomics.

## Introduction

Short insertions and deletions (InDels), as one of the major sources of structural genetic variants, are receiving increasing attention in genetic studies of humans and several model species for their contribution of economic or agricultural traits and human diseases (Mullaney et al., 2010; Yang et al., 2014). In recent years, great efforts have focused on discovering InDel variants, identifying causal InDels, examining the evolutionary processes, and detecting genomic signatures for selection. In model plants *Arabidopsis* and rice, intragenic InDels were correlated to functional roles in flowering time and grain filling (Kuittinen et al., 2008; Rao et al., 2011). Exonic InDels resulting in nonsense mutation or frameshift mutation had also been proved to control carotenoid accumulation in carrot and impair the transcription of gene in pink-fruited tomato (Lin et al., 2014; Iorizzo et al., 2016). All of these studies suggested a potential and important role of InDels in exerting deleterious or beneficial effect on genome and further influencing the phenotypes. However, minimal studies on InDels have been conducted in long-lived perennial and woody plants.

Trees have many notable differences from herbaceous species, particularly in size, lifespan, and perennial and woody growth. They occur in wide geographical distributions that promote abundant diversity in morphology, physiology, adaptation, and disease resistance, which is caused by underlying genetic architectures and their interaction with the environment (Tuskan et al., 2006). Dissecting the causative relationships between naturally occurring allelic variants and phenotypic variation may improve our understanding of the mechanisms of complex quantitative traits, environmental adaptation, and speciation (Mackay et al., 2009). In this regard, progress has been made, primarily through the development and dissection of numerous genome/gene-derived simple sequence repeats (SSRs) and single nucleotide polymorphisms (SNPs) in diverse, natural tree populations (Evans et al., 2014; Du et al., 2015). Recently, InDel variants had been recognized as an effective marker system for genomic studies and molecular marker-assisted breeding applications, and should be employed in genetic studies of trees (Yang et al., 2014).

Moreover, tree growth derives from a succession of developmental process involving cell division and expansion, cell wall deposition and requires the coordinated regulation of diverse biological pathways (Groover, 2005). Stem diameter growth results from cell division activity originating in the vascular cambium where new cells differentiate into phloem that convert photosynthate into water-conducting and supportive xylem tissues (wood). In the past few decades, genetic transformation techniques have helped to make remarkable progress in our understanding of key genes and proteins involved in the biosynthesis of primary and secondary cell wall components (especially lignin) in trees (Groover, 2005). However, these findings also indicate that current methods and understandings are insufficient to fully uncover the complex genetic architecture of cell wall biosynthesis.

Association genetics has proved its power in identification of causal variants underlying quantitative variation in some economically important traits, based on allelic SNPs and/or SSRs (Eckert et al., 2012; McKown et al., 2014); however, “missing heritability” and the low contribution rate explained by each locus (an average effect of c. 5.0% in forest trees) remain unexplained. One frequently assumed explanation is non-SNP allelic variation, for example, InDels (Manolio et al., 2009), which has been largely unexamined for complex traits. Recent progress in *Populus* revealed an alternative approach which captures numerous variants within interacting genes with additive, dominant, and epistatic effects underlying quantitative traits (Du et al., 2015). This novel approach tested the interacting variants and found a highly dynamic and sensitive genetic architecture of complex quantitative traits. It may be suited for identifying the potential gene-derived InDel variants underlying the complexities of wood biosynthesis, in which all investigations on discovery and dissection of InDels remain to be addressed.

Herein, we developed a method that combined RNA-seq and population resequencing profile to identify InDel variants within differentially expressed (DE) genes underlying wood formation in a randomly collection with 435 natural individuals of *Populus tomentosa*. Additionally, we performed multi-InDel association mapping, joining additive, dominant, and epistatic effects to preferably decipher the genetic architecture of growth and lignocellulose biosynthesis in *Populus*, which could provide a better understanding of the causal networks of InDels/genes affecting the quantitative variation in wood formation in trees.

## Materials and Methods

### Population Materials and Phenotyping

The 435 24-year-old, unrelated natural individuals of *P. tomentosa* in this study were from a random collection of 1,047 native individuals (Guan Xian County, Shandong Province, China, 36°23′N, 115°47′E), which represented almost the entire natural distribution range of *P. tomentosa* (**Supplementary Methods S1**). Population structure was estimated and resulted in three climatic regions (Northeastern, Northwestern, and Southern) (Du et al., 2012). Ten growth and wood property traits were measured in this population using at least three replications per phenotype. The growth traits included stem height (*H*), diameter at breast height (DBH), and stem volume (*V*) with at least three technical replications per sample. Wood property traits included three physical properties: fiber length, fiber width (FW), and microfiber angle (MFA), as well as four chemical compositions: holocellulose, α-cellulose, hemicelluloses, and lignin. Details on the sampling method, measuring phenotypic variance, and Pearson’s correlation tests of these traits were described in the previous study (Du et al., 2014).

### Transcriptome Analysis and Identification of DE Genes

Mature xylem, developing xylem, and cambium tissues from a 3-year-old *P. tomentosa* tree (clone “LM50”) were collected at breast height with three biological replicates (**Supplementary Methods S1**). The tissues were immediately frozen in liquid nitrogen and used for high-throughput transcriptome sequencing. Total RNA extraction was performed as Chen et al. (2015) described. The paired-end cDNA library was constructed with the TruSeq RNA Sample Preparation Guide (Illumia) after purifying the total RNA with the RNeasy micro kit (Cat#74004, Qiagen) and then sequenced on Illumina HiSeq 2000 platform to generate 100 bp paired-end reads, following the manufacturer’s instructions (**Supplementary Methods S1**). Three biological replicates were used for all RNA-seq experiments. Raw sequencing data were filtered with the quality requirement using FastX software (v0.0.13, http://hannonlab.cshl.edu/fastx_toolkit/) (**Supplementary Methods S1**). After screening and trimming, all clean reads were mapped to the *Populus trichocarpa* reference genome v3.0 (Sundell et al., 2015) using the splice mapping algorithm in TopHat (v2.0.9, Kim et al., 2013) with default parameters, excepting for multiple hits ≤1. Cufflinks (version 2.1.1, Trapnell et al., 2010) was used to calculate the expression level of genes and DE gene were identified using the fold change (FC) ≥2 or ≤0.5 with the cut off at *P*-value of 1.0e-03 and a *q*-value of 0.10 (**Supplementary Methods S1**).

### Validation of the Expression Patterns of DE Genes by RT-qPCR

To validate the quality of the expression pattern determined from the RNA-seq data, 10 genes were randomly selected from the RNA-seq experiments for tissue-specific expression analysis. Eight tissues and organs from the 3-year-old clone “LM50” of *P. tomentosa*, including root, stem (phloem, cambium, developing xylem, mature xylem), developing leaf, mature leaf, and apical shoot meristem were investigated using real-time quantitative PCR (RT-qPCR). All RT-qPCRs were performed on a 7500 Fast Real-Time PCR System (ABI) using the Light Cycler-Fast Start DNA master SYBR Green I kit (Roche). All primer pairs for the DE genes and the internal control (*Actin*, accession number: EF145577) were designed using Primer Express v3.0 (Applied Biosystems) and are provided in **Supplementary Table S1**. The thermocycler program and reaction components were done as Du et al. (2015) described. All reactions were performed with three technical replications and three biological replications. The data were analyzed using Opticon Monitor Analysis Software v3.1 and standardized to the levels of *Actin* (accession no. GQ988327) using the 2^-ΔΔ*Ct*^ method.

### InDels Calling Based on the Resequencing Data from the *P. tomentosa* Population

All 435 individuals were resequenced with an average of 15-fold coverage (raw data) using the Illumina GA2 instrument and the quality of paired-end short reads of 100 bp was controlled by removing low-quality reads (≥50% of nucleotides with a quality score (*Q*) <20). Then the short reads were mapped and aligned to the *P. trichocarpa* reference genome v3.0 using SOAP aligner/SOAP2 v2.20 with the default options (Li et al., 2008). The mapping rate for different individuals varied from 81 to 92% with the effective mapping depth about 11×. To get high-quality InDels, only the uniquely mapped paired-end reads were used to perform InDels calling, using the GATK v3 (McKenna et al., 2010) with default parameters. The original InDel data was filtered using the Variant Call Format (VCF) tool v4.1 (Danecek et al., 2011) with InDel size >1 bp, quality score (*Q*) > 20, missing rate ≤0.25, and minor allele frequency (MAF) >0.001 (**Supplementary Methods S1**).

InDels for the DE genes were extracted by a custom Python script, including InDels within 2 kb upstream (promoter) and 500 bp downstream (3′UTR). InDel genotypic data for association were filtered by removing: (1) the complex InDels sites (more than two allele at one site); (2) unseparated InDel sites among 435 *P. tomentosa* individuals; (3) minor genotype frequency <5%. After this procedure, the clean InDels and genotypes were obtained for *P. tomentosa* natural population (**Supplementary Methods S1**). Additionally, we validated several of the genic-InDels by PCR and capillary electrophoresis on a capillary sequencer ABI3730xl DNA Analyzer (Applied Biosystems, Carlsbad, CA, United States) and found that the accuracy of genic-InDel calling reached 96.7% (**Supplementary Data S1**).

### InDels *H*_e_ and LD Analysis

InDel heterozygosity (*H*_e_) was calculated by Nei’s expected heterozygosity: $H_{e}=1\,\Sigma p_{t}^{2}$, where *p_t_* represented the allele frequency of the *i*th allele (Nei, 1973). Linkage disequilibrium (LD) tests were performed in the association population using common diallelic InDels (MAF ≥ 0.05). The squared correlation of the allele frequency values (*r*^2^) and high-LD blocks was calculated in Haploview v4.2 (Barrett et al., 2005). To assess the extent of LD, the decay of LD with physical distance was estimated by non-linear regression.

### Association Analysis

#### Single-InDel Analysis

We performed single-InDel association analysis based on the mixed linear model (MLM) in TASSEL v5.0 (Yu et al., 2006), where the population structure matrix (*Q*) and the relative kinship matrix (*K*) were respectively considered to evaluate the effects of population structure and relatedness among individuals. The values of *Q* and *K* were calculated similar to the previous study (Du et al., 2012). A cutoff of positive false discovery rate (FDR) *q* < 0.10 with 10^4^ iterations were used for selecting significance threshold (Storey and Tibshirani, 2003).

#### Analysis of Additive, Dominant, and Epistatic Effects

We used the TASSEL v5.0 to detect the additive and dominant effects, and epistatic effects between each InDel pair was calculated by *epiSNP* computer program (Ma et al., 2008). Here, *epiSNP* is a computer package of serial computing programs, in which an extended Kempthorne model were implemented for estimating epistatic effects between each locus pair. In this program, epistatic effects were orthogonally decomposed divided into four components: additive × additive (A × A), additive × dominance (A × D), dominance × additive (D × A), and dominance × dominance (D × D) interactions, which genetic interpretations of allele × allele, allele × genotype, genotype × allele, and genotype × genotype interactions. More details about this program are described in Ma et al. (2008).

### Transcript Analysis of InDel Genotypes

To test whether the significant InDels (FDR *q* < 0.10) affect the relative transcript abundance of their corresponding DE genes, we quantified the mRNA levels of the different InDel genotypic classes in the association population by RT-qPCR. For each genotypic class, 10 individuals were sampled by obtaining cDNA from the mature xylem, developing xylem, and cambium tissues of the stem at breast height. The differential expression across the three genotypic classes of InDel (SS, SL, and LL represent deletion/deletion, insertion/deletion, and insertion/insertion, respectively) was tested by ANOVA (*P* < 0.01). The specific primer pairs were individually designed for all genes (depending on the positions of significant InDels) and are shown in **Supplementary Table S1**.

## Results

### DE Genes Involved in Wood Formation in *P. tomentosa*

**FIGURE 1:**
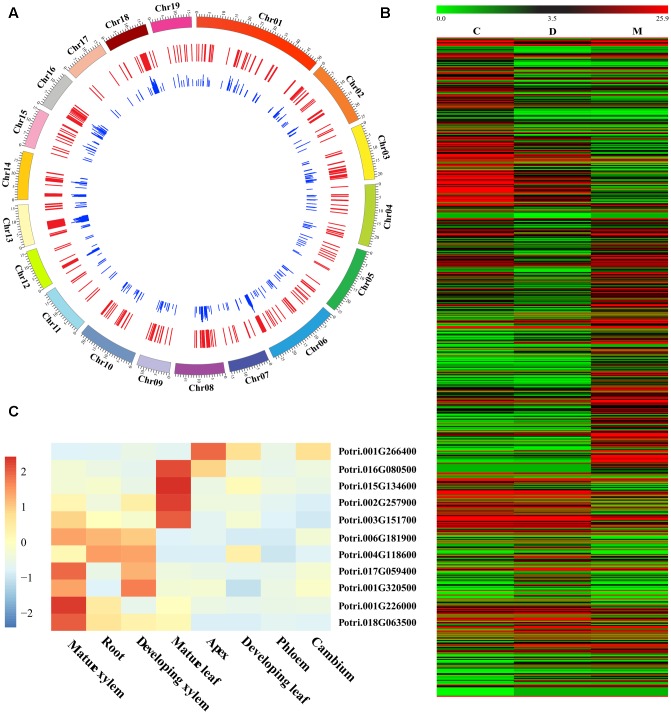
Identification and characterization of differentially expressed (DE) genes in wood tissues of *Populus tomentosa.* **(A)** Circular diagram depicting the genomic distribution of the DE genes. The *P. tomentosa* chromosomes (outer tract), 697 DE genes (middle track), and the frequency of InDels within each gene (inner track) were shown. **(B)** Heat map of transcriptome data for the DE genes in cambium (C), developing xylem (D), and mature xylem (M) tissues. The color scale is shown at the top. **(C)** Relative transcript levels of 10 randomly selected DE genes in different tissues and organs of *P. tomentosa* by RT-qPCR. Expression levels were normalized to the *Actin* gene.

Our RNA-seq comparison of three woody tissues in *P. tomentosa* identified 697 DE genes (FC ≤ 0.5 or ≥2 at *P* < 1.0e-03 with FDR *q* < 0.10, **Figure 1A** and **Supplementary Data S2**). Of these, 322, 122, and 253 showed the highest expression in mature xylem, developing xylem, and cambium tissues, respectively (**Figure 1B**). Furthermore, 10 genes were randomly selected and successfully validated in tissue-specific differential expression by RT-qPCR (**Figure 1C**), suggesting their potential functional role during wood formation.

Combining with resequencing data of *P. tomentosa* and gene models in PopGenie v3.0, a total of 2,565,583 bp sequences for 679 genes/gene fragments were obtained (**Supplementary Data S2**). Gene ontology (GO) analysis revealed 77 significant GO terms in which genes for glucan metabolism, polysaccharide biosynthesis, UDP-glucosyltransferase activity, and molecular binding were over-represented (**Supplementary Data S3**). Functional annotation showed that these genes were related to the cellulose synthase (*CesA*) family proteins, sucrose synthase (*SuSy*), caffeoyl-CoA *O*-methyltransferase (*CCoAOMT*), phenylalanine ammonia-lyase (*PAL*), auxin/indole-3-acetic acid protein (*Aux/IAA*), and auxin response factors (*ARF*). For example, 10 members from *CesA* family showed highest expression in cambium, while only two in mature xylem, suggesting a tissue differential expression of DE genes. We also predicted the motifs present in the promoters of DE genes using PlantPAN 2.0 (Chow et al., 2015), and found several motifs were specifically bound to WRKY, MYB, bHLH, and bZIP transcription factors. All of these strongly suggested DE genes were participated in cellulose and lignin biosynthesis pathway (**Supplementary Data S2**).

### Characterization of Genic InDel Variations in the Natural Population of *P. tomentosa*

**FIGURE 2:**
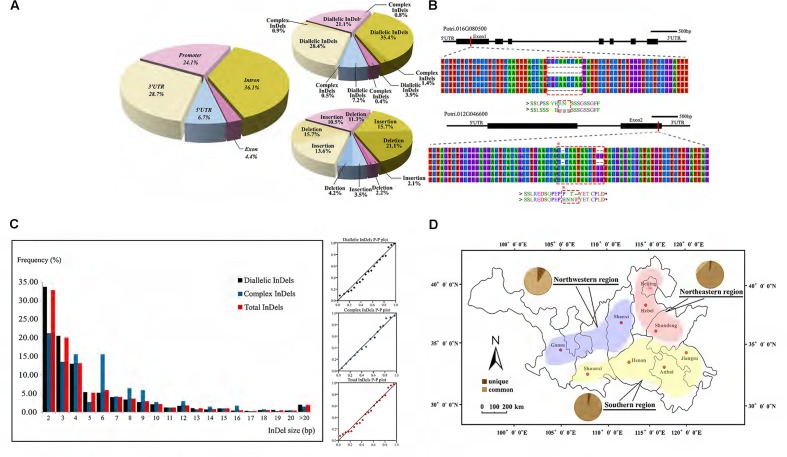
Characterization of genic-InDels in the *P. tomentosa* natural population. **(A)** Distribution pattern of genic-InDels. The distribution of total InDels among the different gene regions was shown on the left. The rates of diallelic/complex InDels and insertions/deletions were shown on the right. **(B)** InDels occurred within exon regions. A deletion of CGCAACCAA (dashed box) in *Potri.016G080500* that caused a deletion of three amino acids [Arg(R)-Asn(N)-Gln(Q)] was shown in the upper figure. Two deletions within *Potri.012G046600* that caused a frameshift (dashed box) were shown in the lower figure. **(C)** The length and distribution frequency of genic-InDels. The frequency distribution of InDels with different sizes (left) and the P–P plot of the frequency distribution trend (right) were shown. **(D)** Distribution patterns of common and unique alleles (pie charts) among the three climatic regions of *P. tomentosa*. The red, blue, and yellow regions represent the northeastern region (NE), the northwestern region (NW), and the southern region (S) of the *P. tomentosa* natural population.

Through the InDel filtering pipeline, 5,482 high-quality InDels, including 2,495 insertions and 2,987 deletions, were identified with an average density of 2.14 InDels per kb and 8 per gene (**Supplementary Table S2** and **Data S4**). Of these, 1,604 were also identified as short tandem repeat. The average length of InDels was 5.0 bp with a range of 2–41 bp. We further characterized the distribution of InDels and found most of them locating in non-coding regions, including promoter (21.9%), intron (36.8%), 5′UTR (7.7%), and 3′UTR (29.4%) (**Figure 2A**). Besides, about 4.3% InDels located in exon where frameshifts or premature termination often results in a putative influence in gene function (**Figure 2B**). Further analysis showed a clearly higher frequency of in-frame InDels, indicating a strong purifying selection on coding regions (**Supplementary Data S5**).

Two major InDels classes were identified by analyzing the sequences of our InDels: diallelic InDels and complex InDels (also defined as multi-allelic InDels) (**Supplementary Table S2**). 5,259 diallelic InDels represented 95.9% of all InDels in our collection from 621 genes with three genotypes of SS, SL, and LL, indicating a dominant position. Among these, the frequency of deletions was slightly higher than that of insertions (deletion/insertion radio was 1.25:1). The majority of discovered InDels (98%) were short (2–20 bp), whereas longer InDels were relatively rare (2.0%), showing a pattern that the frequencies decreased as the length of the InDels increased (**Figure 2C**). Complex InDels only represented 222 sites from 175 genes which generated 731 alleles and varied from three to seven InDels per locus. Specially, 37% multi-allelic sites were caused by containing SNPs in different alleles.

According to the three climatic regions of *P. tomentosa*, unique and common alleles were differentially distributed, and Northwestern region hold the most variable unique alleles (**Figure 2D**). Pathway enrichment analysis of unique alleles revealed enrichment in biosynthesis of secondary metabolites pathway, phenylpropanoid biosynthesis pathway and plant hormone signal transduction pathway, potentially indicating evidence for climate adaptation loci varied across the climatic regions (**Supplementary Data S6**). We further evaluate the InDels *H*_e_ using 742 common diallelic InDels (MAF ≥ 0.05, **Supplementary Data S7**) from 358 unique genes. Results showed the *H*_e_ ranging from 0.19 to 0.50 with an average of 0.44 (**Supplementary Data S8**). In order to study characteristics of pairwise association between diallelic InDels, we performed LD tests among genic InDels. Results showed a similar low LD level (with an average *r*^2^ value of 0.053) which consistent with earlier studies using SNPs. The extent of LD declined rapidly within scales comparable to single gene, however, the levels of LD varied extensively across the genes (**Supplementary Figure S1**).

### Significant Association of InDels Loci with Growth and Wood Property Traits

#### Detection of Single InDel–Trait Associations

In total, 7,420 associations (742 InDels × 10 traits) were performed using the MLM in TASSEL v5.0, which takes into account the kinship matrix and genetic structure (*K* + *Q*), and detected 119 significant associations (FDR *q* < 0.10) representing 89 InDels in 81 DE genes, ranging from one to three for each gene (**Table 1** and **Supplementary Table S3**). Among these, only one (Potri.012G044600_02) was located in exon showing significant association with α-cellulose content. Eighty-two were located in promoter or UTRs, and 36 were located in intron. The individual InDel explained 10.4–24.7% of the phenotypic variation (average *R*^2^ = 14.7%, **Table 1** and **Supplementary Table S3**), implying that these genes might play important roles in tree growth and wood properties. Besides, unique InDels and genes both exhibited significant associations across growth, wood physical and chemical properties. For instance, *Potri.006G251300* (a zinc finger family protein) held two significant InDels which separately associated with lignin, DBH and *V*, suggesting an evidence for potential pleiotropy of allelic loci/genes in tree growth and wood formation, as well as clues for discerning overall genetic effects underlying multi-gene interaction tests.

**Table 1:** Summary of the significant InDel marker associations tested in the *P. tomentosa* natural population with the positive false discovery rate (FDR) *q* < 0.10.

Trait	No. of markers	No. of genes	Average *R*^2^ (%)	Range of *R*^2^ (%)	No. of association
MFA	3	3	14.08	10.89–16.67	3
Fiber length	3	3	13.58	11.78–15.58	3
Fiber width	14	14	15.63	11.03–24.66	14
DBH	32	28	15.62	10.83–23.02	32
*H*	7	7	12.22	10.47–14.66	7
*V*	26	23	14.79	11.09–22.41	26
Hemicellulose	9	8	13.17	11.87–17.75	9
Holocellulose	4	4	13.27	10.41–16.60	4
Lignin	10	10	15.16	12.09–18.67	10
α-Cellulose	11	11	14.71	11.80–19.18	11
Total	89^∗^	81^∗^	14.76	10.41–24.66	119


*^∗^One marker/gene associated with two or more traits. No., number; *R*^2^, percentage of the phenotypic variance explained; MFA, microfiber angle; DBH, diameter at breast height; *H*, stem height; *V*, stem volume; Lignin, lignin content; Holocellulose, holocellulose content; α-Cellulose, α-cellulose content; Hemicellulose, hemicellulose content. Detailed information was showed in **Supplementary Table S3**.*

#### Additive and Dominant Effects of InDels

We further calculated the detailed additive and/or dominant effects for 119 significant associations above (**Table 2**), and 108 associations demonstrated notable additive or dominant effects across 10 traits, representing 74 unique InDels from 68 genes (FDR *q* < 0.10). Of these, nine loci have a combination of additive and dominant effects for a certain trait. Remarkably, two InDels within Potri.008G112200_01 and Potri.008G161200_05 demonstrated opposite direction of dominant effects for different traits, that is, Potri.008G112200_01 associated with MFA for negative dominant effects while with FW for positive dominant effects. Potri.008G161200_05 associated with α-cellulose for positive dominant effects while with *H* for negative dominant effects, indicating a complicated and non-negligible combination effects underlying tree growth and wood biosynthesis, and further implying a combined selection of traits when performing tree breeding.

**Table 2:** Summary of the additive and dominance effects of all significant InDels for each trait in the *P. tomentosa* natural population at a threshold value of FDR *q* < 0.10.

Trait	Additive model				Dominance model			
		
	No. of candidate genes	No. of InDels	Range of effect (%)	Average of *P*-value	No. of candidate genes	No. of InDels	Range of effect (%)	Average of *P*-value
α-Cellulose	6	6	3.17∼8.46	5.09E-03	6	6	-10.74∼9.79	3.62E-03
Hemicellulose	6	6	3.66∼5.52	1.86E-03	3	3	-6.86∼-8.53	2.58E-03
Holocellulose	3	3	4.09∼5.08	2.66E-03	/	/	/	/
Lignin	4	4	1.07∼2.18	1.17E-03	4	4	1.70∼2.47	1.52E-03
Fiber length	1	1	0.05	5.94E-04	2	2	-0.13∼0.04	3.19E-03
Fiber width	3	3	0.97∼1.98	3.60E-03	9	9	-2.01∼3.40	3.56E-03
MFA	1	1	3.14	6.39E-04	3	3	-3.65∼-2.50	3.41E-03
*H*	4	4	1.12∼1.94	2.34E-03	2	2	-2.16∼-1.80	2.96E-03
DBH	22	25	2.15∼5.08	1.65E-03	4	4	-4.62∼4.40	2.74E-03
*V*	17	19	0.15∼0.34	2.22E-03	3	3	-0.28∼-0.25	2.05E-03
Total	68	74	0.05∼8.46	2.21E-03	32	32	-10.74∼9.79	2.98E-03


*No., number; *P*-value, significant level for association; MFA, microfiber angle; DBH, diameter at breast height; *H*, stem height; *V*, stem volume; Lignin, lignin content; Holocellulose, holocellulose content; α-Cellulose, α-cellulose content; Hemicellulose, hemicellulose content. Detailed information was showed in **Supplementary Table S4**.*

Furthermore, analysis of the pattern of gene–trait–effect revealed four association groups: (1) when a gene only associated with one trait, the gene showed single effect. For instance, the gene *Potri.003G058600* (coding a *Populus* hypothetical protein) only associated with MFA for dominant effect. However, as clues for potential pleiotropy, genes always appeared to have multi-trait associations, which resulted in following three complicated groups. (2) A gene represented the same effects for all associated traits. For example, *Potri.014G106600* (similar to caffeic acid/5-hydroxyconiferaldehyde *O*-methyltransferase, *COMT*) showed all additive effects for DBH, α-cellulose, and *V*. (3) A gene showed separated additive or dominance effects for different traits. For example, *Potri.013G154700* (similar to expansin S1 precursor, *EXP*) showed an additive effect for DBH but a dominant effect for FW. (4) A gene showed a combination of additive and dominant effects. For example, *Potri.008G161200* (similar to aux/IAA protein, *PtIAA14.1*) showed only additive effect for *V* whereas a combination of additive and dominant effects for *H* and α-cellulose content (**Supplementary Table S4**). These findings implied that when genes function in separate metabolic pathway which generating different traits, they might perform diverse or even opposite effects, and then these pathways overlapped or shared common systemic signals finally resulting in complicated biological networks and variable phenotypes.

#### Identification of Significant InDels Using Genotypic Differential Expression

**FIGURE 3:**
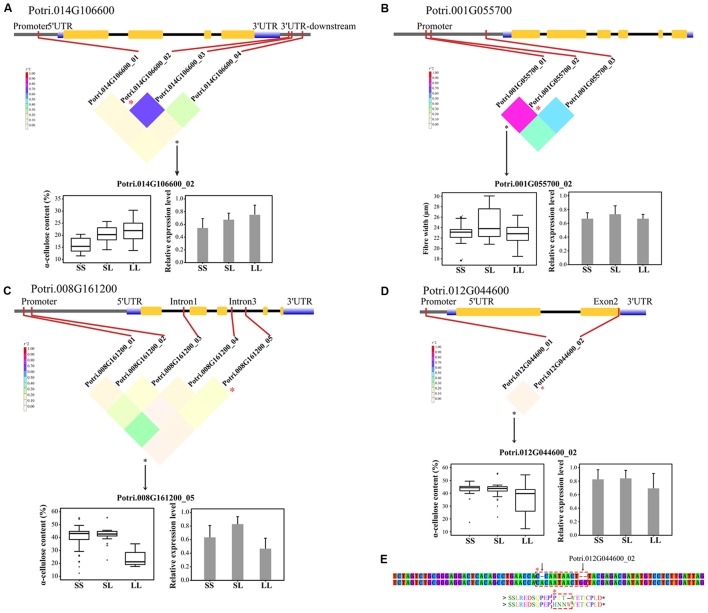
Dissection of the regulatory InDels with additive and/or dominant effects in the *P. tomentosa* natural population. **(A)** A causal InDel (Potri.014G106600_02, T > TATTA) within the 3′UTR-downstream of *Potri.014G106600* (*COMT*) showed a significant association with α-cellulose under additive effect. **(B)** A causal InDels (Potri.001G055700_02, A > AACCT) within the promoter region of *Potri.001G055700* (*4CL*) showed significant association with fiber width under dominant effect. **(C)** A causal InDel (Potri.008G161200_05, ATATATATAT > A) within the intron of *Potri.008G161200* (similar to the indole-3-acetic acid protein, *IAA*) showed a significant association with α-cellulose contents under additive and dominant effects. **(D)** A causal InDel (Potri.016G090300_02, T > TGG) within exon 2 of *Potri.012G044600* (a receptor-like kinase) showed a significant association with α-cellulose content under additive effect. **(E)** Potri.016G090300_02, together with another 1-bp insertion (C > CA), caused an ORF shift and an amino acid insertion (dashed box). SS, SL, and LL represent deletion/deletion, insertion/deletion, and insertion/insertion, respectively. The colored, inverted triangle represents the linkage disequilibrium (LD) levels (the color of each box corresponds to the legend). The genotypic effects on phenotypic traits (box plots) and the relative expression level (histograms) are shown.

To validate the significant associated InDels, we quantified the relative abundance of mRNA products for the corresponding genes among the three genotypic classes of each significant InDel. In total, 74 tests (74 unique InDels, **Table 2**) representing 68 genes indicated that only seven genes showed significant differential expression among their genotypic classes at *P* < 0.01 (**Supplementary Table S5**). Most of the significant InDels were located in non-coding regions where InDels could be attributed to the differences noted in genotype-specific gene expression (**Figures 3A–C** and **Supplementary Table S5**). In addition, the significant InDels located in exon may also change the transcript levels of their target genes (**Figures 3D,E**).

### Epistatic Interactions of InDels and DE Genes in the Natural Population of *P. tomentosa*

Potential epistatic interactions of InDels within DE genes for growth and wood property traits were examined by epiSNP package using all InDels. In total, 1,004 significant InDel epistatic pairs (*P* < 1e-04, *q* < 0.10) were detected with four decomposed components of A × A, A × D, D × A, and D × D (**Supplementary Figure S2**), representing 517 InDels from 290 unique genes (**Table 3** and **Supplementary Data S9**). The significant epistatic interacted InDels covered 69.6% of the tested InDels, in which only 58 (11.3%) displayed significant additive/dominant effect (**Table 3**), implying a possibility that epistatic interactions commonly occurred between mutations that without significant main effects.

**Table 3:** Summary of the additive, dominant, and epistatic effects of all significant InDels for each trait in *P. tomentosa* natural population.

Trait	No. of interacting InDels^a^	Epistatic pairs (InDel–InDel)	No. of genes	Epistatic pairs (gene–gene)	No. of interacting InDels under additive and dominance	No. of genes harboring three genetic effects^b^
α-Cellulose	142	117	115	115	18	18
Hemicellulose	121	94	97	92	11	11
Holocellulose	146	140	117	136	24	21
Lignose	85	55	74	54	10	10
Fiber length	110	94	93	92	11	10
Fiber width	115	87	95	87	17	17
MFA	77	50	66	50	9	9
*H*	42	23	38	23	4	4
DBH	94	63	84	62	10	10
*V*	207	279	164	273	30	28
Total	517	940	290	906	58	53


*No., number; MFA, microfiber angle; DBH, diameter at breast height; *H*, stem height; *V*, stem volume; Lignin, lignin content; Holocellulose, holocellulose content; α-Cellulose, α-cellulose content; Hemicellulose, hemicellulose content. ^a^The significance threshold of *P* ≤ 1E-04 under epistasis. ^b^The number of significant genes showing additive, dominance, and epistatic effects for each trait.*

**FIGURE 4:**
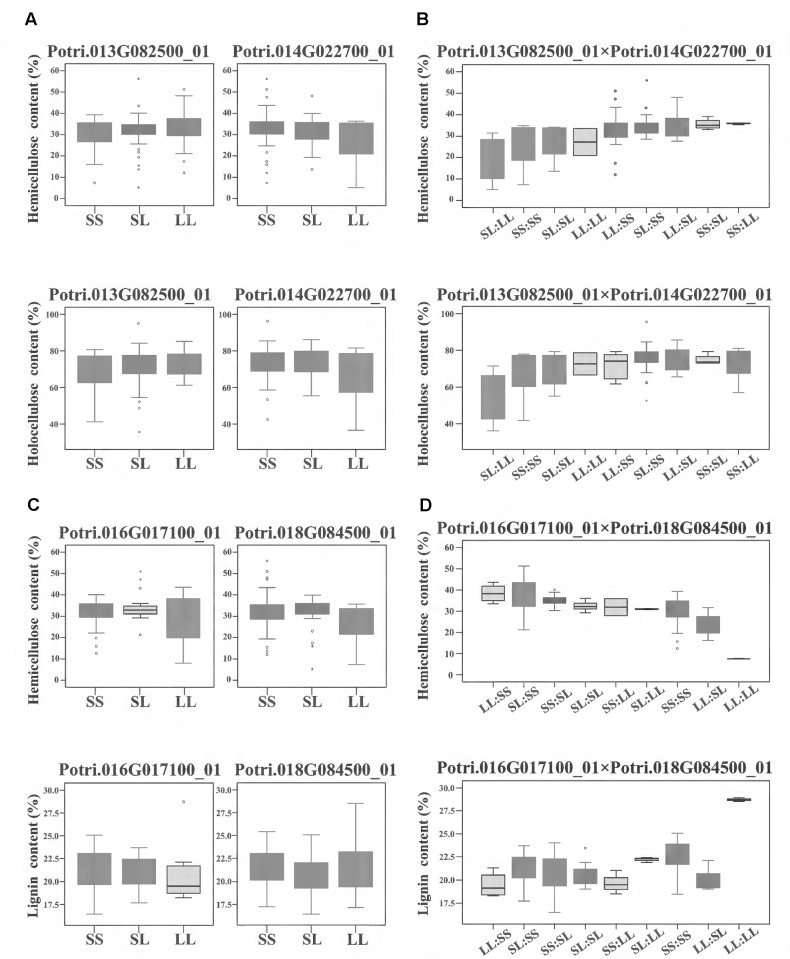
The epistatic interactions of InDel pairs in the *P. tomentosa* natural population. **(A,B)** The epistatic interaction between Potri.013G082500_01 and Potri.014G022700_01 showed a negative D × A effect for hemicellulose and holocellulose contents. For hemicellulose contents (upper line), in the absence of epistasis, genotypic effects for Potri.013G082500_01 and Potri.014G022700_01 were almost same. However, with epistasis, their genotypic combination effect (SS:LL) leaded to a considerable increase in hemicellulose contents (an average increase of 18%) and the combination of SL:LL leaded to approximately a 38% decrease in hemicellulose contents. For holocellulose contents (lower line), similar genotypic effects were found for the genotypic combination. **(C,D)** The epistatic interaction between Potri.016G017100_01 and Potri.018G084500_01 showed an opposite A × A effect on hemicellulose content (upper line) and lignin contents (lower line). In the absence of epistasis, the genotypic effects for Potri.016G017100_01 and Potri.018G084500_01 were almost same. However, with epistasis, the genotypic combination LL:LL led to the highest lignin content as well as the lowest hemicellulose content, and the genotypic effect for hemicellulose content dropped with the increase of lignin content. **(A,C)** Box plot indicates the additive/dominant effect for each InDel without epistatic effects. **(B,D)** Box plot indicates the epistatic effects between InDel pairs. D × A and A × A represent dominance × additive and additive × additive interaction. SS, SL, and LL represent deletion/deletion, insertion/deletion, and insertion/insertion, respectively.

To further investigate how the epistatic interactions affecting different traits, we identified 122 epistatic InDel pairs which associated with at least two traits. For instance, in the model of calculating additive/dominant effect, Potri.013G082500_01 and Potri.014G022700_01 did not display any significant associations, whereas under the epistatic model, their combinations of different genotypes showed unexpected non-additive effects on hemicellulose and holocellulose contents with synergetic effect (**Figures 4A,B**). Another epistatic InDel pair, between Potri.016G017100_01 and Potri.018G084500_01 (without main effect), showed an antagonistic effect with lignin and hemicellulose contents (**Figures 4C,D**). This revealed a pattern that the same combinations of genotypes under epistatic effect could either enhance or reduce different traits at the same time, strongly suggested that epistasis gave a considerable and complicated influence on phenotypic variations even though without additive/dominant effect (main effect).

**FIGURE 5:**
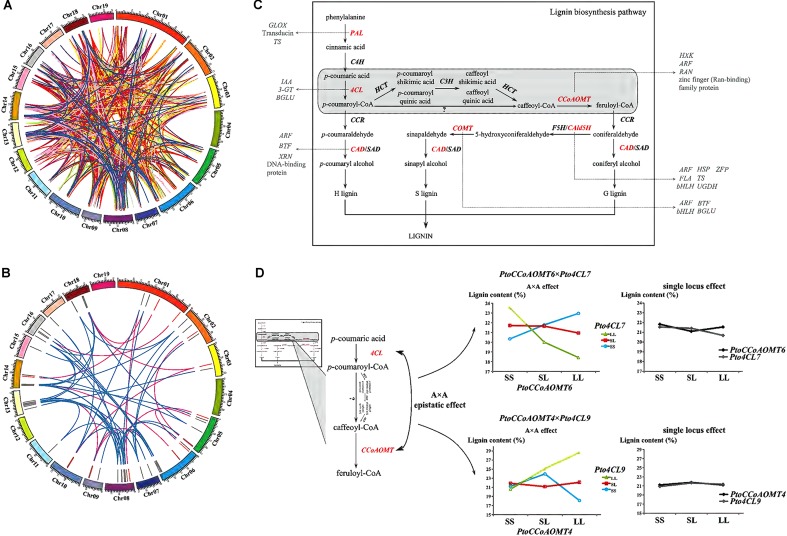
Dissection of the gene–gene epistatic interaction networks for 10 growth and wood-property traits. **(A)** Circular diagram depicting 973 gene–gene interaction networks for 10 growth and wood-property traits. The different colored links represent different traits. Detailed information for each trait was showed in **Supplementary Figure S3**. **(B)** Circular diagram depicting the 55 epistatic gene–gene interaction networks underlying lignin contents. The outer track showed the *P. Tomentosa* chromosomes (Chr01 through Chr19 on an Mb scale). The middle track showed the epistatic genes involved in the monolignol biosynthesis pathway (red) or in other pathways (gray). The inner links showed the epistatic interactions between genes where at least one gene belonged to the monolignol biosynthesis pathway (pink), or neither belonged (blue). **(C)** Proposed pathway of lignin biosynthesis. The genes that had epistatic effects involved in the lignin biosynthesis pathway (red), and the putative genes and epistatic interactions that contributed to the proposed monolignol biosynthesis pathway revealed in our study (gray) were shown. **(D)** Dissection of two epistatic gene–gene interactions underlying the monolignol biosynthesis pathway with A × A epistatic effects. PAL, phenylalanine ammonia-lyase; C4H, cinnamate-4-hydroxylase; 4CL, 4-coumarate:CoA ligase; CCR, cinnamoyl-CoA reductase; CAD, cinnamyl alcohol dehydrogenase; SAD, sinapyl alcohol dehydrogenase; HCT, *p*-hydroxycinnamoyl-CoA:quinateshikimate *p*-hydroxycinnamoyltransferase; C3H, 4-coumarate 3-hydroxylase; CCoAOMT, caffeoyl-CoA *O*-methyltransferase; CAld5H/F5H, coniferyl aldehyde 5-hydroxylase; COMT, caffeic acid/5-hydroxyconiferaldehyde *O*-methyltransferase; 3-GT, anthocyanidin 3-*O*-glucosyltransferase; ARF, auxin responsive factor; BGLU, beta glucosidase; BTF, basic transcription factor; FLA, FASCICLIN-like arabinogalactan protein precursor; GLOX, glyoxal oxidase; bHLH, basic helix-loop-helix transcription factor; HSP, heat shock protein; HXK, hexokinase; IAA, indole-3-acetic acid; RAN, RAN GTPase; TS, threonine synthase; UGDH, UDP-glucose dehydrogenase; XRN, exoribonuclease; ZFP, zinc finger protein.

For improving our understanding of the genetic architecture affecting complex phenotypic variation, the gene–gene interaction networks for each trait were drawn based on the epistatic InDel pairs between chromosomes (918 pairs) and within the chromosomes (86 pairs) (**Figure 5A** and **Supplementary Figure S3**). In total, 973 gene–gene interactions were detected and 127 of these interactions were associated with at least two traits (**Supplementary Figure S3**). Furthermore, we focused on 55 gene–gene epistatic interactions underlying lignin content to detect the potential epistatic networks affecting lignin biosynthesis (**Figure 5B**). Among these interactions, only 12 genes were mapped to monolignol biosynthesis pathway (Shi et al., 2010), while majority of the remaining genes were belonged to other biologic pathways, including cellulose synthesis and hormone metabolism (**Figure 5C**). Specifically, two interactions between four lignin-related genes, *PtoCCoAOMT6* with *Pto4CL7* (4-coumarate:CoA ligase), and *PtoCCoAOMT4* with *Pto4CL9*, were observed having opposite epistatic effects, both of which showed A × A epistatic effects consistent with the proposed monolignol biosynthesis pathway (**Figure 5D** and **Supplementary Table S6**). When ignoring the epistatic effect, the phenotypic effects of lignin content for the different genotypes at these four genes were almost the same (no main effect). However, in the presence of epistasis, the lignin content at three genotypic classes of *PtoCCoAOMT6* was decreased at *Pto4CL7* by LL and increased at *Pto4CL7* by SS, which contributed to their negative epistatic effects (**Supplementary Table S6**). Similarly, with positive epistatic effects, the lignin content at three genotypic classes of *PtoCCoAOMT4* was sharply enhanced at *Pto4CL9* by LL and SS, but this increasing trend was suppressed by the genotype combination of LL at *PtoCCoAOMT4* and SS at *Pto4CL9*.

## Discussion

### Identification and Characterization of InDels within Candidate Genes Involved in Wood Formation in *Populus*

InDels had been recognized as the second most abundant form of genetic variation in humans (Mullaney et al., 2010) and widely distributed among several annual species (Kuittinen et al., 2008; Rao et al., 2011; Yang et al., 2014). Here, combining transcriptome sequencing with population resequencing programs, we identified a set of DE genes involved in wood formation with 5,841 InDel polymorphisms had a genome-wide representation through *P. tomentosa*, a perennial woody species (**Figure 1A**). Approximately 95.9% of these InDels were diallelic InDels and their length and distribution showed a similar pattern to that of the total InDels (**Figure 2C**), indicating their dominant position at the gene level. Complex InDels were generally excluded in many earlier studies because of limited statistical and methodological methods. While combining GATK v3 with PCR and capillary electrophoresis (**Supplementary Data S1**), we found 222 (3.8%) complex InDels from 175 genes which generated 731 alleles (**Figure 2C**), 37% of these were caused by containing SNPs in different alleles. It has been assumed that complex InDels provide a large proportion of the inherited susceptibility to inflammatory bowel disease (McGovern et al., 2005), suggesting that such complex InDels might influence biological traits and deserve more comprehensive investigation. Interestingly, we also observed co-separation of InDels with nearby SNPs. Recently, InDels and SNPs within the same haplotype block were identified in carrot which resulted into high carotenoid accumulation in carrot (Iorizzo et al., 2016), as well as InDel and SNP variants had similar LD profiles (Lu et al., 2012), thus implying inclusion of InDels with other gene-derived genetic markers to reveal a more detailed picture of the sequence variation for genomics-assisted breeding applications in woody plants.

The influence of gene function due to InDels was expected to be greater than that of substitutions as they gave rise to a more severe alteration in the sequence. As consequence, the InDels identified in this study were mapped to both coding and non-coding regions, showing significantly lower frequency and dominating in-frame InDels in coding regions (**Figure 2A** and **Supplementary Data S5**). This result is similar to findings in recent studies on the distribution of genic-SNPs and SSRs in *Populus*, although the density of InDels was lower than the other two genic markers (Du et al., 2015). It also suggests stronger purifying selection within the coding regions and that exon-derived InDels may have more deleterious effects as they can disrupt protein structure as well as influence transcriptional or post-transcriptional regulation (**Figures 2B**, **3D,E**; Iorizzo et al., 2016). Here, 4.3% of the InDels were found in coding regions (**Figure 2A**), which may contribute to alterations of gene function. Moreover, InDels (21.9%) located in promoter may envisioned to explain the differences in gene expression and have large effects on phenotypic variation in *Populus* (**Figures 3A–C** and **Supplementary Table S5**).

The decay of LD for InDels within genes was similar to our recent findings based on SNPs within 11 candidate genes involved in cellulose biosynthesis (Du et al., 2015), highlighting the promising utility of small InDels in LD-based association mapping (**Supplementary Figure S1**). However, the extent of LD varied extensively across genes, which might reflect alterable natural selection strength on different parts of genome. Collectively, our findings suggest that InDels could be a valuable complement to sequence variances such as SNPs for improving the marker density and resolutions in genome-wide association mapping studies (Lu et al., 2012).

### Functional Interpretations of InDel–Trait Associations in the Natural Population of *P. tomentosa*

Our study provides an in-depth genetic dissection of gene-derived InDels in a natural *Populus* population under additive, dominance and epistasis models. Most importantly, this study, being the first InDel-based association mapping study on a long-lived perennial species, sheds light on detecting more causal genes/variations underlying quantitative traits. Interestingly, the majority of the InDels detected by the single-InDel association model had a higher effect size (average of 14.7%, **Table 1**) than that of c. 5.0% explained by the single-SNP association model in poplar species (McKown et al., 2014; Du et al., 2015). Even though such large phenotypic variance probably results from some other potentially linked markers, due to the alteration in the sequence, InDels are likely to have the major impact on phenotypic variance, which highlights an important role in the dissection of quantitative traits and molecular breeding.

Here, we detected an InDels located in exon region that led to ORF shifts and exhibited significant associations with wood properties (**Figures 3D,E**). In detail, Potri.012G044600_02, a 2-bp insertion (T > TGG) located within exon 2 of *Potri.012G044600* (similar to receptor-like kinase) was significantly associated with α-cellulose content. Further analysis revealed another 1-bp insertion (C > CA) which was just eight bp upstream of Potri.012G044600_02. These two sites simultaneously showed an ORF shift and an amino acid (AA) insertion [Pro(P)-Ile(I)-Thr(T) > His(H)-Asn(N)-Asn(N)-Trp(W); **Figures 3D,E**], suggesting that the locus might be in close proximity to the causal polymorphisms or even the functional variant itself. Analysis of protein structure revealed that this ORF shift (PIT > HNNW) was 16 AAs downstream of the serine/threonine/tyrosine protein kinase (STYKc) domain, suggesting a possible effect on the protein kinase domain for genetic regulation of α-cellulose content. This conjecture was also supported by the significant differences in expression among the three genotypic classes of Potri.012G044600_02 (**Figure 3D**). Therefore, these results suggest that InDels, especially the exon-InDels, could potentially affect gene function and finally result in phenotypic variation.

Lignin is polymerized mainly from coniferyl (G) and sinapyl (S) as well as low level of *p*-coumaryl (H) in *Populus*, which synthesized by phenylpropanoid pathway with key enzymes like PAL, 4CL, cinnamyl alcohol dehydrogenase (CAD), and CCoAOMT (Shi et al., 2010). The biosynthesis pathway also regulated by some well-known transcription factors belonging to NAC, MYB, and WRKY gene families (Vanholme et al., 2010). Here, two non-coding InDels, Potri.009G095800_01 and Potri.013G067500_05 were found to significantly associate with lignin content under additive/dominant effects (**Supplementary Tables S4**, **S5**). *Potri.009G095800* was belonging to CAD family that catalyze the last step of reducing hydroxyl cinnamyl aldehydes to their corresponding monolignols (Shi et al., 2010). Previous studies reported that deficiency of *CAD* led to abnormal lignin structural changes (Ralph et al., 1997). Supportively, the significant differences in expression of *PtCAD4.1* were found among the three genotypic classes (**Supplementary Table S5**), suggesting that InDels might have a regulatory effect or are in very strong LD with a nearby regulatory polymorphism (Thumma et al., 2009). Moreover, *Potri.013G067500* was similar to MYB transcription factor that had been demonstrated diverse functions in lignin biosynthesis pathway. Collectively, our findings show that significant InDels/genes associating with specific traits were validated by direct biochemical pathway of genes, and also suggested that InDels could change traits from altering the expression of gene in certainly biological pathways (Shi et al., 2010).

Biosynthesis of cell wall components is coordinated with other biological processes during plant vascular development and these genes are possibly involved or indirectly participate in the shared pathways (Eckert et al., 2012). For instance, two loci (Potri.007G076500_03 and Potri.007G076500_04) close to the 3′UTR of *CesA* (*Potri.007G076500*) were associated with hemicellulose content. The encoded protein is predominantly essential for building a cellulose-hemicellulose network, which creates strong but flexible plant cell walls (Cosgrove, 2005). Previous research also showed the significant haplotypes within *CesA* were also associated with hemicellulose (Du et al., 2015), suggesting that some cellulose synthase genes might be involved into hemicellulose biosynthesis. Contrasting with the known genes or biochemical links in biological pathways, our study also identified significant genes/variants of unknown function (**Supplementary Table S4**), suggesting that genetic associations could not only uncover candidate genes involved in shared pathways, but also reveal genes which have not yet been placed within known biological pathways. These findings provide valuable clues for understanding the biology inside the “black box” that lies between genotype and phenotype in terms of causal networks of interacting genes.

Genetic association studies often had an interesting observation that a genetic locus/gene is associated with multiple, sometimes seemingly distinct traits, consisting with our results (**Supplementary Tables S3**, **S4**). Such associations highlight the potential that traits share the common genetic pathways and underscore the relevance of pleiotropy (Solovieff et al., 2013). However, trait correlations can also result in such associations while no causal relation exists (Platt et al., 2010). When measuring the target traits at the population level, the resulting phenotypes would inevitably be correlated because of that traits had to be hierarchical refined into multifactorial components. In our previous report, correlation matrices of all 10 traits were estimated (Du et al., 2014), implying that the correlated traits used here tended to be biologically related and shared the genetic bases (Porth et al., 2013). Recently, a multi-trait mixed model considering correlated phenotypes in structured populations has been reported (Korte et al., 2012), provided insight into distinguishing the pleiotropy loci from statistical associations. However, this model just focused on relatively simple pairwise correlations between two traits, challenges were still exist for larger multi-traits experiments.

### Epistasis of InDels for Association Studies in the *P. tomentosa* Natural Population

In quantitative genetics, epistasis is defined as the statistical interaction between genotypes at two or more loci and also refers to a modification of the additive or dominant effects of the interacting loci. Therefore, phenotypic variations cannot be predicted simply by summing the effects of individual loci (Mackay, 2014). Epistasis is an important component of the genetic basis of complex traits, though often ignored because estimation of epistatic interactions has been challenging due to small population sizes, limited statistical methods, and the high computational demands. In this study, we detected large numbers of significant InDel–InDel epistatic pairs associated with tree growth and wood properties, covering c. 70% of all investigated InDels (**Figure 5A** and **Table 3**). This result revealed a pervasive epistatic effect among allelic mutations in a natural population of *Populus*, which was consistent with observations in *Drosophila*, mice, *Arabidopsis*, yeast, and humans (Flint and Mackay, 2009; Huang et al., 2012), and provides essential information for a deeper understanding of the mechanisms of gene interactions in this perennial species.

Here, we observed that only a small part of the InDels (11.3%) within the epistatic interactions showed significant additive and/or dominant effects, and epistasis showed variable effects on different phenotypes even with the same combinations of genotypes (**Figure 4** and **Table 3**). This observation further supports the idea that epistasis plays a key role in genetic architecture and has a considerable influence on phenotypic variations when constricting to main effect (Mackay, 2014). In addition, some investigations have shown that epistasis could be responsible for the missing heritability and the lack of replication in association genetics (Manolio et al., 2009). Epistatic interactions in populations depend on allele frequencies, and therefore, the replication of estimating loci with epistatic effects would be different among diverse populations, but the underlying epistatic architecture in different populations seems to be the same (Huang et al., 2012). Consequently, our detection of epistasis could contribute to the quantitative variation, which was hidden or not apparent in the identification of the main effects and could potentially improve genetic predictions in modern plant breeding programs.

Two pairwise InDel interactions detected for lignin content illustrated the ability of epistasis for dissecting underlying genetic networks (**Supplementary Table S6** and **Figure 5D**). Supporting this, two interactions were both occurred between *CCoAOMT* and *4CL*, which matched the step of proposed monolignol biosynthesis pathway (Shi et al., 2010). These results demonstrated that epistasis could not only change the direction of phenotypic effects, but also alter its magnitude (Mackay, 2014), in which the phenotypic effect of one locus was either enhanced (negative effect for *PtoCCoAOMT6* and *Pto4CL7*) or suppressed (positive effect for *PtoCCoAOMT4* and *Pto4CL9*) by the other locus (**Figure 5D**). It was also supported by our transcriptome data that enhanced pairwise showed consistent expression in xylem tissue, whereas suppressed pairwise exhibited opposite case (**Supplementary Figure S4**). In addition, epistatic interaction between genes where the biological evidence for how they interacted had not yet been found were identified (**Figures 4A**, **5B,C**). This further illuminated that the genetic architecture of each quantitative trait was controlled by the interactions of multiple genes that belong to diverse biological pathways, suggesting that the power of epistasis could be as important as additive/dominant effects. However, we only detected the pairwise interactions, three- or even higher-order interactions between loci have not yet been explored, as estimating the exponential number of possible interactions at these levels remains a challenge.

## Conclusion

In conclusion, we showed the first investigation of the InDel variants underlying the complexities of quantitative traits in a long-lived perennial species, which enables closer examination of the number and effect magnitudes of functionally relevant trait-regulatory genes/alleles responsible for complex quantitative traits in plants. We also showed valuable utility of InDels that may have roles in the dissection of quantitative traits and molecular breeding. Epistatic interactions of InDels suggested a powerful role in potentially improving genetic predictions in modern plant breeding programs. Finally, our findings will enable an effective marker system for molecular marker-assisted breeding applications, and further facilitate our understanding of quantitative genomics.

## Data Archiving

The accession numbers of all genes in the GenBank Data Library: KU573093 to KU573771; All data corresponding to genes, InDels and genotypes used in this study were provided as Supplementary Data.

## Author Contributions

DZ planned and designed the research. CG, QD, JX,-48pc MQ, and BC performed the experiments. CG, QD, and DZ analyzed the data. CG wrote the paper. All authors read and approved the manuscript.

## Conflict of Interest Statement

The authors declare that the research was conducted in the absence of any commercial or financial relationships that could be construed as a potential conflict of interest.

## Supplementary Material

**FIGURE S1 |** Linkage disequilibrium (LD) in the *P. tomentosa* natural population.

**FIGURE S2 |** Representatives of the four components of epistatic effects.

**FIGURE S3 |** Detailed information of the gene–gene interaction networks for 10 growth and wood property traits.

**FIGURE S4 |** Heatmap of the transcriptome data for four epistatically interacting genes involved in the proposed monolignol biosynthesis pathway.

**TABLE S1 |** The RT-qPCR primers used in this study.

**TABLE S2 |** Summary of the 5,482 InDels identified within 629 genes or gene fragments after filtering pipeline.

**TABLE S3 |** Detailed information on significant InDel marker associations tested in the *P. tomentosa* natural population using the single-InDel model with a positive false discovery rate (FDR) *q* < 0.10.

**TABLE S4 |** Detailed information on the additive and dominance effects of all significant InDels for each trait in the *P. tomentosa* natural population at a threshold value of FDR *q* < 0.10.

**TABLE S5 |** Summary of seven significant differentially expressed genes among three genotypic classes at *P* < 0.01.

**TABLE S6 |** Two epistatic gene–gene interactions underlying the monolignol biosynthesis pathway with A × A epistatic effects between four lignin-related genes.

**DATA S1 |** Validation of InDels by PCR amplification and capillary electrophoresis.

**DATA S2 |** Summary of 697 differentially expressed genes with an FDR *q* < 0.10 and *P* < 1.0e-03.

**DATA S3 |** Seventy-seven significant gene ontology (GO) terms of 679 genes or gene fragments identified from the *P. tomentosa* genomic resequencing data with an FDR-adjusted value of ≤0.05.

**DATA S4 |** Detailed information on the 5,482 high-quality InDels identified from the 629 genes or gene fragments with a quality score (*Q*) >20.

**DATA S5 |** Frequency distribution of InDels sizes within coding regions.

**DATA S6 |** Pathway enrichment analysis of unique alleles among three climatic regions.

**DATA S7 |** The genotypic data for 742 common InDels (minor allele frequency ≥0.05) used in the association analysis of the *P. tomentosa* natural population.

**DATA S8 |** Detailed InDel heterozygosity calculated by Nei’s expected heterozygosity.

**DATA S9 |** Detailed information on the significant pairwise epistatic effects among the InDels for each trait in the *P. tomentosa* natural population with *P* < 1e-04.
