# Supplementary material for: Dissection of Insertion–Deletion Variants within Differentially Expressed Genes Involved in Wood Formation in Populus
Source: Front Plant Sci. 2018 Jan 18;8:2199. doi: 10.3389/fpls.2017.02199 (PMC5778123; doi:10.3389/fpls.2017.02199)
Supplement: Supplementary file 5 [file Table_1.DOC]

**Table S1** The RT-qPCR primers used in this study

| **Gene model** | **Forward primer(5′→3′ )** | **Forward primer(5′→3′ )** |
| --- | --- | --- |
| Potri.001G055700 | TGGCGTCCAGGTTCAAGAAG | GCCTTTACTAGGATCCCCATGA |
| Potri.001G199100 | CCCACCACAAAACCAGAACTTC | CGTCGAGGGAGTAGGACTCTGT |
| Potri.001G226100 | CCTCCACGGAGCCTTACATAGT | AGCGGGCATTGAGTGTTACC |
| Potri.001G266400 | GTTCCCTAAGTGGCTCCCT | GACGGTTCTCCTTCATTGTCATA |
| Potri.001G372400 | GGTGGGTGCCCAAAGTCTT | GCCATGCTTCCTGACACTAGCT |
| Potri.001G453600 | CAGGTTTCGCAGGAGATGATG | CATCTTTCTGCCCCATTCCA |
| Potri.002G114200 | GGCCGAGACAAATCACGTAAC | GAGCCGAGAGAGACCATTGTG |
| Potri.002G197000 | GCACGGAGAGACTTGGAAATTT | CCCAGCCACGAGTTTTTTTTAT |
| Potri.003G058600 | GCTCTGGAGCAAACACATCTGA | CCCTGGACCTGTGTTGAACTC |
| Potri.003G099700 | CGTGGCCTCAACACTGAAAAAT | CCCCAGGAGGCAATAAAGC |
| Potri.003G142300 | AATTCCGATCACCGCATACG | GGCTCTGAGACCTTTGGGAAA |
| Potri.004G051600 | TTGCCACAGAACCCCTTACAC | TGCCTCCAATATGCGAATTTG |
| Potri.004G228800 | CAACGCGGACGAGACCTTTA | CTCGGAAGCCAAGAGAATTGA |
| Potri.006G024300 | CAAGCTCGCTCCTTTGAACCT | ACCGCAAAAGTCCAGCATTT |
| Potri.006G127500 | CAAATTTTTCCGATGGCTCTGT | TTTCGGTGCTTGTCCTTGCT |
| Potri.006G251300 | GGAAGTTTCGGTGGAAGATCAT | CAGCCGTGCAATACCAATCA |
| Potri.006G257100 | AAGGTTGAAACCGGTGATTCTC | CCATCACCAGAAGACCCAAAA |
| Potri.006G270700 | TCAGGAAAGGGAAAGCCTACAA | CCGTATCCCAAAAGGTCAAATG |
| Potri.007G016400 | TTCGCCGAAGATTGCCATAT | TGGTCCATCATGTGCATGCT |
| Potri.007G076500 | TGACTGGTGGAGAAATGAGCAA | GCTGACACCTGCCAAAACCT |
| Potri.008G080800 | GCAAGACTAATGGCATCGATGA | TCCAGCAGCATTACCTCCAAA |
| Potri.008G082100 | GGCGAACGATGTCATAAATGTG | CCCGACAGTGAGATAAGCAATG |
| Potri.008G089900 | GCAGGATGGTTGGCTAAGCA | CGAGGGAACCATCCAAGGT |
| Potri.008G094000 | GCCCATTTCCCAGGAGCTA | GGATCTTCAGACGAGCGTTCA |
| Potri.008G094700 | GAGTTGGTGAGACATGCCGATA | CCCCATGGATGTCAAGTGCTA |
| Potri.008G097600 | TTCCAGGCCGCAGTTCTTC | ACCTGCTTGGCGAGTTCAAT |
| Potri.008G112200 | CGCCAAACAATCCAAAAGGT | TGAACCCTGTCTCCCCAACA |
| Potri.008G116500 | GCCGGAGCAACTGGTTTTATC | CAGGGCGGACAAGAGCATA |
| Potri.008G118300 | TCCTCACGACGATGCTGTTG | GGCCCAATACTCGTGTTCAAG |
| Potri.008G161200 | TTCCATGGGAGATGTTTGTCAA | CGGTGCTGAAACAAGTCCAA |
| Potri.009G095800 | TGTAGGATGGGCAGCAACAG | TGGCCCCGTATCTCTGAGACT |
| Potri.009G123600 | GCTTCATGTCGTGGCTGTGT | GGCCTATTCGCAAAGACAACA |
| Potri.010G099700 | TGTGTTTTGGTTTGTGGTTCTT | CCAAACGACCCATATCTCAGT |
| Potri.010G100200 | GCACTGTTCATGGGCATAGCT | CCCCCTGGTCTCTGAAATCTG |
| Potri.011G059300 | CGGCCAATGGAGGGATAGT | TGCACGACACCAAATCTTGACT |
| Potri.011G148000 | ATCGGTGCAGAGCGTTTCC | TTTCGTGAATTCCAGGTGCTT |
| Potri.011G148200 | CGCAGATGAAGGCCTTGTACA | CACCAGCAGTCTTCAAAAGCAA |
| Potri.012G037300 | TGGTCAGTGGAGCATTGCATA | AACGATGCAAGAAACAGCAAGA |
| Potri.012G037900 | ACCTGCAATCGAGGAAGTCAA | CCACTGACAACCCATGTGTTG |
| Potri.012G040600 | GAACGGAGGTTTTCACCATAGG | GGCAACATATTTGGAGGCTTCT |
| Potri.012G044600 | TTGGTCCTCCCTCCACTCCTA | CATCAGCCTTTTGGGAAACC |
| Potri.013G064700 | GACGGAGGTTGGAACATGGA | TCTGCAAGCTCATCCCATACTG |
| Potri.013G066000 | TGGGTTTCGATTCGTTGGA | GAAGCAGCAGAGGGAGAAACAG |
| Potri.013G067000 | CATCTCATTCCCTGTTCCCTTT | AGGGTTTGCTTTGCTTGTTTG |
| Potri.013G067500 | CAGCAAAAGTTGCAGGCTTAGA | GCTGCCCATTTGTTACCCAAT |
| Potri.013G068700 | AGAAAGGAGAGCTGGCATGATT | CGAGCTCGCCTAATTTTCACTT |
| Potri.013G068900 | CCTCAGTTTTTGCCCAACATC | TCTGCGGTCCATGTCATTGA |
| Potri.013G070200 | ATAACTGCCGACGTCAAGGAA | ATCGTTAGGGTTAGGGCAGGAA |
| Potri.013G078500 | TCAGCCTAGGCCTGGTAAAGG | GCAAGAGAGCAGAAGCTGAATG |
| Potri.013G154700 | GCAATCCTCCTCTCCAACATTT | TCCAGCTCGGTATTGAGCAA |
| Potri.014G016300 | CTGGAGCTTTGGGTCTGAACA | TCTGCACCAAAAGCAATGCT |
| Potri.014G017700 | GAACTCGCATCCCAAGAGCTA | TGATTATCCCATGAGGGTTTCC |
| Potri.014G018200 | CCAGATGAAGGTCAGGAACACA | CCGGCTCTTGGTTGAAGGT |
| Potri.014G022400 | CAGCGACCGTGTCAGATGAGT | GAGGGTGCGAAGACTGACATTC |
| Potri.014G106600 | AGCAGGTCCAGATGCCAAAC | GGTGCACCTGGGTTCTTTGT |
| Potri.014G120700 | TCACCTTCCCTCGACAATATCA | CCAGCTATTGTTGCCATTCTTG |
| Potri.014G121000 | TCGAGTCTTCATGCCCTTGTT | TGAGGTTCCCCTTTGTGTTGT |
| Potri.014G121900 | TCGAGCCAATGACGATTAGAGA | GCGGTGGCTCTTTCAGCTAT |
| Potri.014G123000 | ACTTCACATAGCTCCGCCAAA | ACAACACGCGATAGCTCAGTGT |
| Potri.015G073800 | ACCGATCCCAGAAGAAACGA | GGGAAGAAAAACAGGGAAAACA |
| Potri.016G013700 | CTTGAGGAGGTGGAAGGAACAG | GGCTTTCCATTCTCAGGAACTG |
| Potri.016G066300 | CCCAGTCAAAATCCGAAATCC | AAGAGGAGGAGGCCAGAAAAGA |
| Potri.016G068200 | GGAAGTCCCGTGCAAAAGC | CAAGAAACGGCCACCACATC |
| Potri.016G090300 | CGGACTGCCTTTTGAGACTGT | TGAACCGCATCCCTGAACA |
| Potri.017G014400 | GGCAACAACAGCTGAGAAAAGA | GCTTAGGGACAGGGACTGGTT |
| Potri.018G028300 | GGTGGTGGCTTTTGTATTTGC | GCCAAACGATAGTCAACGGAAA |
| Potri.018G098900 | TGCCTAGTCATGTTGTATCTTT | CAAGTGTCTGACAGAAATTACAC |
| Potri.018G145800 | AGCACTCATGGAGGTTTCT | GGCGGGAATGTCTTTTC |
| Potri.001G055700 | TGGCGTCCAGGTTCAAGAAG | GCCTTTACTAGGATCCCCATGA |
| Potri.001G199100 | CCCACCACAAAACCAGAACTTC | CGTCGAGGGAGTAGGACTCTGT |
| Potri.001G226100 | CCTCCACGGAGCCTTACATAGT | AGCGGGCATTGAGTGTTACC |
| Potri.001G266400 | GTTCCCTAAGTGGCTCCCT | GACGGTTCTCCTTCATTGTCATA |
| Potri.001G372400 | GGTGGGTGCCCAAAGTCTT | GCCATGCTTCCTGACACTAGCT |
| Potri.001G453600 | CAGGTTTCGCAGGAGATGATG | CATCTTTCTGCCCCATTCCA |
| Potri.002G114200 | GGCCGAGACAAATCACGTAAC | GAGCCGAGAGAGACCATTGTG |
| Potri.002G197000 | GCACGGAGAGACTTGGAAATTT | CCCAGCCACGAGTTTTTTTTAT |
| Potri.003G058600 | GCTCTGGAGCAAACACATCTGA | CCCTGGACCTGTGTTGAACTC |
| Potri.003G099700 | CGTGGCCTCAACACTGAAAAAT | CCCCAGGAGGCAATAAAGC |
| Potri.003G142300 | AATTCCGATCACCGCATACG | GGCTCTGAGACCTTTGGGAAA |
| Potri.004G051600 | TTGCCACAGAACCCCTTACAC | TGCCTCCAATATGCGAATTTG |
| Potri.004G228800 | CAACGCGGACGAGACCTTTA | CTCGGAAGCCAAGAGAATTGA |
| Potri.006G024300 | CAAGCTCGCTCCTTTGAACCT | ACCGCAAAAGTCCAGCATTT |
| Potri.006G127500 | CAAATTTTTCCGATGGCTCTGT | TTTCGGTGCTTGTCCTTGCT |
| Potri.006G251300 | GGAAGTTTCGGTGGAAGATCAT | CAGCCGTGCAATACCAATCA |
| Potri.006G257100 | AAGGTTGAAACCGGTGATTCTC | CCATCACCAGAAGACCCAAAA |
| Potri.006G270700 | TCAGGAAAGGGAAAGCCTACAA | CCGTATCCCAAAAGGTCAAATG |
| Potri.007G016400 | TTCGCCGAAGATTGCCATAT | TGGTCCATCATGTGCATGCT |
| Potri.007G076500 | TGACTGGTGGAGAAATGAGCAA | GCTGACACCTGCCAAAACCT |
| Potri.008G080800 | GCAAGACTAATGGCATCGATGA | TCCAGCAGCATTACCTCCAAA |
| Potri.008G082100 | GGCGAACGATGTCATAAATGTG | CCCGACAGTGAGATAAGCAATG |
| Potri.008G089900 | GCAGGATGGTTGGCTAAGCA | CGAGGGAACCATCCAAGGT |
| Potri.008G094000 | GCCCATTTCCCAGGAGCTA | GGATCTTCAGACGAGCGTTCA |
| Potri.008G094700 | GAGTTGGTGAGACATGCCGATA | CCCCATGGATGTCAAGTGCTA |
| Potri.008G097600 | TTCCAGGCCGCAGTTCTTC | ACCTGCTTGGCGAGTTCAAT |
| Potri.008G112200 | CGCCAAACAATCCAAAAGGT | TGAACCCTGTCTCCCCAACA |
| Potri.008G116500 | GCCGGAGCAACTGGTTTTATC | CAGGGCGGACAAGAGCATA |
| Potri.008G118300 | TCCTCACGACGATGCTGTTG | GGCCCAATACTCGTGTTCAAG |
| Potri.008G161200 | TTCCATGGGAGATGTTTGTCAA | CGGTGCTGAAACAAGTCCAA |
| Potri.009G095800 | TGTAGGATGGGCAGCAACAG | TGGCCCCGTATCTCTGAGACT |
| Potri.009G123600 | GCTTCATGTCGTGGCTGTGT | GGCCTATTCGCAAAGACAACA |
| Potri.010G099700 | TGTGTTTTGGTTTGTGGTTCTT | CCAAACGACCCATATCTCAGT |
| Potri.010G100200 | GCACTGTTCATGGGCATAGCT | CCCCCTGGTCTCTGAAATCTG |
| Potri.011G059300 | CGGCCAATGGAGGGATAGT | TGCACGACACCAAATCTTGACT |
| Potri.011G148000 | ATCGGTGCAGAGCGTTTCC | TTTCGTGAATTCCAGGTGCTT |
| Potri.011G148200 | CGCAGATGAAGGCCTTGTACA | CACCAGCAGTCTTCAAAAGCAA |
| Potri.012G037300 | TGGTCAGTGGAGCATTGCATA | AACGATGCAAGAAACAGCAAGA |
| Potri.012G037900 | ACCTGCAATCGAGGAAGTCAA | CCACTGACAACCCATGTGTTG |
| Potri.012G040600 | GAACGGAGGTTTTCACCATAGG | GGCAACATATTTGGAGGCTTCT |
| Potri.012G044600 | TTGGTCCTCCCTCCACTCCTA | CATCAGCCTTTTGGGAAACC |
| Potri.013G064700 | GACGGAGGTTGGAACATGGA | TCTGCAAGCTCATCCCATACTG |
| Potri.013G066000 | TGGGTTTCGATTCGTTGGA | GAAGCAGCAGAGGGAGAAACAG |
| Potri.013G067000 | CATCTCATTCCCTGTTCCCTTT | AGGGTTTGCTTTGCTTGTTTG |
| Potri.013G067500 | CAGCAAAAGTTGCAGGCTTAGA | GCTGCCCATTTGTTACCCAAT |
| Potri.013G068700 | AGAAAGGAGAGCTGGCATGATT | CGAGCTCGCCTAATTTTCACTT |
| Potri.013G068900 | CCTCAGTTTTTGCCCAACATC | TCTGCGGTCCATGTCATTGA |
| Potri.013G070200 | ATAACTGCCGACGTCAAGGAA | ATCGTTAGGGTTAGGGCAGGAA |
| Potri.013G078500 | TCAGCCTAGGCCTGGTAAAGG | GCAAGAGAGCAGAAGCTGAATG |
| Potri.013G154700 | GCAATCCTCCTCTCCAACATTT | TCCAGCTCGGTATTGAGCAA |
| Potri.014G016300 | CTGGAGCTTTGGGTCTGAACA | TCTGCACCAAAAGCAATGCT |
| Potri.014G017700 | GAACTCGCATCCCAAGAGCTA | TGATTATCCCATGAGGGTTTCC |
| Potri.014G018200 | CCAGATGAAGGTCAGGAACACA | CCGGCTCTTGGTTGAAGGT |
| Potri.014G022400 | CAGCGACCGTGTCAGATGAGT | GAGGGTGCGAAGACTGACATTC |
| Potri.014G106600 | AGCAGGTCCAGATGCCAAAC | GGTGCACCTGGGTTCTTTGT |
| Potri.014G120700 | TCACCTTCCCTCGACAATATCA | CCAGCTATTGTTGCCATTCTTG |
| Potri.014G121000 | TCGAGTCTTCATGCCCTTGTT | TGAGGTTCCCCTTTGTGTTGT |
| Potri.014G121900 | TCGAGCCAATGACGATTAGAGA | GCGGTGGCTCTTTCAGCTAT |
| Potri.014G123000 | ACTTCACATAGCTCCGCCAAA | ACAACACGCGATAGCTCAGTGT |
| Potri.015G073800 | ACCGATCCCAGAAGAAACGA | GGGAAGAAAAACAGGGAAAACA |
| Potri.016G013700 | CTTGAGGAGGTGGAAGGAACAG | GGCTTTCCATTCTCAGGAACTG |
| Potri.016G066300 | CCCAGTCAAAATCCGAAATCC | AAGAGGAGGAGGCCAGAAAAGA |
| Potri.016G068200 | GGAAGTCCCGTGCAAAAGC | CAAGAAACGGCCACCACATC |
| Potri.016G090300 | CGGACTGCCTTTTGAGACTGT | TGAACCGCATCCCTGAACA |
| Potri.017G014400 | GGCAACAACAGCTGAGAAAAGA | GCTTAGGGACAGGGACTGGTT |
| Actin | CTCCATCATGAAATGCGATG | TTGGGGCTAGTGCTGAGATT |
