# Supplementary material for: Dissection of Insertion–Deletion Variants within Differentially Expressed Genes Involved in Wood Formation in Populus
Source: Front Plant Sci. 2018 Jan 18;8:2199. doi: 10.3389/fpls.2017.02199 (PMC5778123; doi:10.3389/fpls.2017.02199)
Supplement: Supplementary file 6 [file Table_2.doc]

**Table S2** Summary of the 5,482 InDels identified within 629 genes or gene fragments after filtering pipeline

| **InDels** | **No. of InDel sites** | **No. of Insertion** | **No. of Deletion** | **No. of genes** |
| --- | --- | --- | --- | --- |
| Diallele InDel | 5,259 | 2,328 | 2,931 | 621 |
| Complex InDel* | 223 | 167 | 56 | 175 |
| Total | 5,482 | 2,495 | 2,987 | 629 |

No.= number.

* Complex InDel defined as an InDel with more than two alleles at one site.
