# Supplementary material for: Dissection of Insertion–Deletion Variants within Differentially Expressed Genes Involved in Wood Formation in Populus
Source: Front Plant Sci. 2018 Jan 18;8:2199. doi: 10.3389/fpls.2017.02199 (PMC5778123; doi:10.3389/fpls.2017.02199)
Supplement: Supplementary file 7 [file Table_3.DOC]

**Table S3** Detailed information on significant InDel marker associations tested in the *P. tomentosa* natural population using the single-InDel model with a positive false discovery rate (FDR) *q* < 0.10

| **Trait** | **Marker** | **Gene model** | **Location** | **Ref allele** | **Alt alleles** | ***P*** | ***R*^2^(%)** |
| --- | --- | --- | --- | --- | --- | --- | --- |
| α-cellulose | Potri.002G114200_02 | Potri.002G114200 | 3'UTR-downstream | CATG | C | 2.29E-03 | 14.36 |
| α-cellulose | Potri.004G228800_03 | Potri.004G228800 | intron | AGAC | A | 2.76E-03 | 13.46 |
| α-cellulose | Potri.006G257100_02 | Potri.006G257100 | 3'UTR-downstream | A | AAAAATCT | 9.43E-03 | 12.96 |
| α-cellulose | Potri.008G161200_05 | Potri.008G161200 | intron | ATATATATAT | A | 2.04E-04 | 18.62 |
| α-cellulose | Potri.010G111400_01 | Potri.010G111400 | promoter | T | TTC | 4.32E-03 | 13.37 |
| α-cellulose | Potri.012G037900_02 | Potri.012G037900 | 3'UTR-downstream | G | GTTTAAGTTTTAAGTCTTAAGT | 3.21E-03 | 13.74 |
| α-cellulose | Potri.012G044600_02 | Potri.012G044600 | exon | T | TGG | 2.05E-03 | 15.95 |
| α-cellulose | Potri.013G068900_01 | Potri.013G068900 | 3'UTR-downstream | TTTC | T | 8.10E-04 | 19.18 |
| α-cellulose | Potri.014G106600_02 | Potri.014G106600 | 3'UTR-downstream | T | TATTA | 2.95E-03 | 13.25 |
| α-cellulose | Potri.014G123000_02 | Potri.014G123000 | intron | A | ATAT | 1.37E-03 | 15.14 |
| α-cellulose | Potri.018G028300_01 | Potri.018G028300 | promoter | TTCA | T | 5.70E-03 | 11.80 |
| MFA | Potri.003G058600_01 | Potri.003G058600 | promoter | AGACATGCATATG | A | 9.75E-03 | 10.89 |
| MFA | Potri.008G112200_01 | Potri.008G112200 | promoter | AGC | A | 8.03E-04 | 16.67 |
| MFA | Potri.014G018200_01 | Potri.014G018200 | promoter | C | CCTATG | 1.86E-03 | 14.67 |
| DBH | Potri.001G199100_01 | Potri.001G199100 | 3'UTR-downstream | A | AGT | 8.07E-03 | 11.34 |
| DBH | Potri.001G226100_01 | Potri.001G226100 | promoter | C | CCA | 4.23E-03 | 15.04 |
| DBH | Potri.001G266400_01 | Potri.001G266400 | intron within 5'UTR | T | TTTTTTTTTATTTTAGA | 4.78E-03 | 14.33 |
| DBH | Potri.002G197000_01 | Potri.002G197000 | promoter | G | GAGTATTCA | 8.63E-03 | 11.15 |
| DBH | Potri.004G051600_01 | Potri.004G051600 | 3'UTR-downstream | GCCTCC | G | 5.77E-04 | 18.00 |
| DBH | Potri.005G146900_01 | Potri.005G146900 | intron | AAAT | A | 1.50E-03 | 15.15 |
| DBH | Potri.005G236700_03 | Potri.005G236700 | promoter | TTGTGTGTGTGTGTG | TTGTGTGTGTGTGTGTGTG | 1.12E-03 | 15.57 |
| DBH | Potri.006G004200_01 | Potri.006G004200 | promoter | G | GGCC | 2.81E-03 | 13.61 |
| DBH | Potri.006G024300_04 | Potri.006G024300 | 3'UTR | AAG | A | 6.10E-04 | 20.69 |
| DBH | Potri.006G127500_01 | Potri.006G127500 | 3'UTR-downstream | A | AGGATTAT | 5.10E-05 | 22.06 |
| DBH | Potri.006G251300_04 | Potri.006G251300 | promoter | G | GAT | 2.38E-03 | 15.23 |
| DBH | Potri.007G016400_01 | Potri.007G016400 | intron | AAC | A | 2.25E-03 | 14.11 |
| DBH | Potri.008G082100_01 | Potri.008G082100 | 3'UTR | TGCG | T | 4.97E-03 | 12.32 |
| DBH | Potri.008G094700_02 | Potri.008G094700 | 3'UTR | A | AAG | 9.65E-03 | 11.32 |
| DBH | Potri.008G094700_05 | Potri.008G094700 | 5'UTR | GACAATCA | G | 3.64E-03 | 13.07 |
| DBH | Potri.008G097600_04 | Potri.008G097600 | intron | C | CTA | 7.28E-03 | 14.87 |
| DBH | Potri.008G116500_01 | Potri.008G116500 | 3'UTR-downstream | C | CCT | 5.36E-05 | 22.70 |
| DBH | Potri.012G040600_01 | Potri.012G040600 | promoter | TTC | T | 1.76E-03 | 14.95 |
| DBH | Potri.013G066000_02 | Potri.013G066000 | promoter | TTC | T | 5.00E-04 | 17.45 |
| DBH | Potri.013G067000_01 | Potri.013G067000 | 3'UTR-downstream | CTTTTTA | C | 1.78E-03 | 15.28 |
| DBH | Potri.013G154700_01 | Potri.013G154700 | intron | A | ACT | 4.75E-03 | 12.79 |
| DBH | Potri.014G017700_01 | Potri.014G017700 | 3'UTR | A | ACT | 9.27E-03 | 12.66 |
| DBH | Potri.014G106600_01 | Potri.014G106600 | promoter | GTAA | G | 2.09E-03 | 15.05 |
| DBH | Potri.014G106600_02 | Potri.014G106600 | 3'UTR-downstream | T | TATTA | 3.60E-03 | 13.02 |
| DBH | Potri.014G120700_01 | Potri.014G120700 | 3'UTR | A | AAAG | 1.31E-03 | 15.28 |
| DBH | Potri.014G121000_01 | Potri.014G121000 | promoter | T | TAA | 3.16E-05 | 23.02 |
| DBH | Potri.017G014400_02 | Potri.017G014400 | promoter | ACAGG | A | 9.76E-03 | 10.83 |
| DBH | Potri.018G028300_01 | Potri.018G028300 | promoter | TTCA | T | 9.05E-04 | 16.04 |
| DBH | Potri.018G030000_03 | Potri.018G030000 | intron | CTT | C | 4.17E-04 | 17.84 |
| DBH | Potri.018G145800_02 | Potri.018G145800 | intron | TTGA | T | 2.75E-04 | 18.51 |
| DBH | Potri.018G145800_03 | Potri.018G145800 | intron | A | AAT | 2.99E-04 | 18.33 |
| DBH | Potri.018G145800_04 | Potri.018G145800 | intron | C | CAATG | 3.65E-04 | 18.11 |
| Fibre length | Potri.001G372400_01 | Potri.001G372400 | intron | A | ATAT | 1.57E-03 | 15.58 |
| Fibre length | Potri.008G118300_01 | Potri.008G118300 | promoter | TTTTG | T | 2.59E-03 | 13.38 |
| Fibre length | Potri.016G013700_01 | Potri.016G013700 | promoter | CCATTTA | C | 8.44E-03 | 11.78 |
| Fibre width | Potri.001G001500_04 | Potri.001G001500 | promoter | A | AAAAT | 1.94E-03 | 17.16 |
| Fibre width | Potri.001G055700_02 | Potri.001G055700 | promoter | A | AACCT | 1.40E-03 | 16.24 |
| Fibre width | Potri.002G227300_02 | Potri.002G227300 | intron | G | GTTAA | 9.37E-03 | 11.53 |
| Fibre width | Potri.003G099700_02 | Potri.003G099700 | 3'UTR-downstream | C | CAAACT | 1.80E-04 | 23.58 |
| Fibre width | Potri.008G096400_01 | Potri.008G096400 | promoter | ATGCATTTGTCTTGTTACCGCT | A | 4.89E-03 | 13.56 |
| Fibre width | Potri.008G112200_01 | Potri.008G112200 | promoter | AGC | A | 5.40E-03 | 12.21 |
| Fibre width | Potri.010G099700_01 | Potri.010G099700 | 3'UTR-downstream | TTA | T | 3.66E-03 | 12.93 |
| Fibre width | Potri.011G148000_01 | Potri.011G148000 | 3'UTR-downstream | G | GGTAC | 2.50E-03 | 16.26 |
| Fibre width | Potri.011G148200_01 | Potri.011G148200 | intron | T | TGGATATC | 6.40E-04 | 19.39 |
| Fibre width | Potri.012G037300_01 | Potri.012G037300 | 3'UTR-downstream | CTAAT | C | 6.44E-05 | 24.66 |
| Fibre width | Potri.013G154700_01 | Potri.013G154700 | intron | A | ACT | 9.59E-03 | 11.03 |
| Fibre width | Potri.014G022400_01 | Potri.014G022400 | promoter | G | GCTGTGGTC | 5.34E-03 | 12.14 |
| Fibre width | Potri.014G025300_01 | Potri.014G025300 | intron | A | AATT | 5.26E-03 | 13.82 |
| Fibre width | Potri.014G121900_01 | Potri.014G121900 | intron | C | CTTG | 2.22E-03 | 14.38 |
| H | Potri.003G142300_02 | Potri.003G142300 | intron | T | TTA | 3.64E-03 | 14.66 |
| H | Potri.004G230900_02 | Potri.004G230900 | intron | GGT | G | 9.62E-03 | 10.47 |
| H | Potri.006G270700_06 | Potri.006G270700 | intron | TTTATG | T | 7.11E-03 | 11.26 |
| H | Potri.008G080800_06 | Potri.008G080800 | 3'UTR | TTTTCGCCCTCATCTAGTC | T | 7.67E-03 | 10.77 |
| H | Potri.008G161200_05 | Potri.008G161200 | intron | ATATATATAT | A | 2.40E-03 | 13.29 |
| H | Potri.014G016300_01 | Potri.014G016300 | 5'UTR | T | TCC | 4.38E-03 | 12.23 |
| H | Potri.018G082800_03 | Potri.018G082800 | 3'UTR-downstream | TAA | T | 8.70E-03 | 12.85 |
| Hemicellulose | Potri.001G453600_01 | Potri.001G453600 | promoter | G | GCTT | 3.63E-03 | 12.67 |
| Hemicellulose | Potri.007G076500_03 | Potri.007G076500 | 3'UTR-downstream | C | CCGG | 2.23E-03 | 13.85 |
| Hemicellulose | Potri.007G076500_04 | Potri.007G076500 | 3'UTR-downstream | G | GTTTTT | 5.29E-03 | 11.93 |
| Hemicellulose | Potri.008G089900_02 | Potri.008G089900 | intron | G | GTT | 5.52E-03 | 11.87 |
| Hemicellulose | Potri.009G123600_01 | Potri.009G123600 | promoter | A | AAAAT | 3.59E-03 | 13.13 |
| Hemicellulose | Potri.012G037900_02 | Potri.012G037900 | 3'UTR-downstream | G | GTTTAAGTTTTAAGTCTTAAGT | 6.33E-03 | 12.29 |
| Hemicellulose | Potri.015G073800_02 | Potri.015G073800 | intron | TAAA | T | 1.33E-03 | 17.75 |
| Hemicellulose | Potri.016G068200_02 | Potri.016G068200 | intron | CAAGTAGTG | C | 6.31E-03 | 12.64 |
| Hemicellulose | Potri.016G090300_02 | Potri.016G090300 | 3'UTR | GGAAAA | G | 6.37E-03 | 12.44 |
| Holocellulose | Potri.001G199100_02 | Potri.001G199100 | 3'UTR | AAAAC | A | 8.63E-03 | 10.41 |
| Holocellulose | Potri.001G453600_03 | Potri.001G453600 | promoter | A | AGC | 3.91E-03 | 13.66 |
| Holocellulose | Potri.010G100200_05 | Potri.010G100200 | promoter | AAAAAAAAAG | A | 7.42E-03 | 12.41 |
| Holocellulose | Potri.016G066300_01 | Potri.016G066300 | promoter | CGTCAAAACAGGTTAACCT | C | 3.69E-03 | 16.60 |
| Lignin | Potri.006G251300_02 | Potri.006G251300 | promoter | CAA | C | 5.32E-04 | 17.25 |
| Lignin | Potri.008G082100_01 | Potri.008G082100 | 3'UTR | TGCG | T | 2.56E-04 | 18.35 |
| Lignin | Potri.008G118300_01 | Potri.008G118300 | promoter | TTTTG | T | 2.48E-03 | 13.82 |
| Lignin | Potri.009G095800_01 | Potri.009G095800 | intron | T | TTAC | 2.60E-03 | 13.57 |
| Lignin | Potri.011G059300_02 | Potri.011G059300 | promoter | T | TAG | 4.49E-04 | 17.96 |
| Lignin | Potri.013G019800_03 | Potri.013G019800 | 3'UTR-downstream | T | TCGGATTAATG | 7.90E-03 | 12.80 |
| Lignin | Potri.013G067500_05 | Potri.013G067500 | 3'UTR-downstream | GTTTTTTGTTTTTTC | G | 1.16E-03 | 18.67 |
| Lignin | Potri.013G070200_03 | Potri.013G070200 | 3'UTR-downstream | T | TAA | 8.94E-03 | 12.09 |
| Lignin | Potri.013G078500_02 | Potri.013G078500 | intron | TATCCCAGTACCCCC | T | 3.09E-03 | 14.02 |
| Lignin | Potri.018G098900_01 | Potri.018G098900 | intron | T | TATAAAC | 3.82E-03 | 13.08 |
| V | Potri.001G266400_01 | Potri.001G266400 | intron within 5'UTR | T | TTTTTTTTTATTTTAGA | 9.81E-03 | 12.53 |
| V | Potri.004G051600_01 | Potri.004G051600 | 3'UTR-downstream | GCCTCC | G | 7.20E-03 | 12.28 |
| V | Potri.005G146900_01 | Potri.005G146900 | intron | AAAT | A | 1.78E-03 | 14.80 |
| V | Potri.005G236700_03 | Potri.005G236700 | promoter | TTGTGTGTGTGTGTG | TTGTGTGTGTGTGTGTGTG | 1.14E-03 | 15.56 |
| V | Potri.006G024300_04 | Potri.006G024300 | 3'UTR | AAG | A | 2.46E-03 | 17.34 |
| V | Potri.006G127500_01 | Potri.006G127500 | 3'UTR-downstream | A | AGGATTAT | 5.32E-05 | 22.03 |
| V | Potri.006G251300_04 | Potri.006G251300 | promoter | G | GAT | 7.87E-03 | 12.42 |
| V | Potri.008G094000_05 | Potri.008G094000 | 3'UTR-downstream | GGGGA | G | 3.42E-03 | 13.18 |
| V | Potri.008G094700_02 | Potri.008G094700 | 3'UTR | A | AAG | 7.86E-03 | 11.87 |
| V | Potri.008G116500_01 | Potri.008G116500 | 3'UTR-downstream | C | CCT | 9.29E-04 | 16.74 |
| V | Potri.008G161200_05 | Potri.008G161200 | intron | ATATATATAT | A | 5.23E-04 | 17.13 |
| V | Potri.012G040600_01 | Potri.012G040600 | promoter | TTC | T | 5.03E-05 | 22.41 |
| V | Potri.013G064700_02 | Potri.013G064700 | intron | TTC | T | 1.89E-03 | 14.85 |
| V | Potri.013G066000_02 | Potri.013G066000 | promoter | TTC | T | 2.62E-03 | 13.94 |
| V | Potri.013G067000_01 | Potri.013G067000 | 3'UTR-downstream | CTTTTTA | C | 9.06E-03 | 11.63 |
| V | Potri.013G068700_01 | Potri.013G068700 | intron | T | TAAAAGAAAAG | 9.05E-03 | 11.09 |
| V | Potri.013G070900_01 | Potri.013G070900 | intron | T | TTTA | 8.21E-03 | 11.31 |
| V | Potri.014G017700_01 | Potri.014G017700 | 3'UTR | A | ACT | 1.71E-03 | 16.82 |
| V | Potri.014G106600_01 | Potri.014G106600 | promoter | GTAA | G | 5.17E-03 | 13.14 |
| V | Potri.014G106600_02 | Potri.014G106600 | 3'UTR-downstream | T | TATTA | 8.55E-03 | 11.22 |
| V | Potri.014G121000_01 | Potri.014G121000 | promoter | T | TAA | 3.87E-04 | 18.00 |
| V | Potri.018G028300_01 | Potri.018G028300 | promoter | TTCA | T | 1.49E-03 | 15.00 |
| V | Potri.018G030000_03 | Potri.018G030000 | intron | CTT | C | 1.81E-03 | 14.75 |
| V | Potri.018G145800_02 | Potri.018G145800 | intron | TTGA | T | 9.63E-04 | 15.93 |
| V | Potri.018G145800_03 | Potri.018G145800 | intron | A | AAT | 1.14E-03 | 15.57 |
| V | Potri.018G145800_04 | Potri.018G145800 | intron | C | CAATG | 3.98E-03 | 13.02 |

*P*-value = significant level for association.

*R*^2^ = percentage of the phenotypic variance explained.

MFA = microfiber angle; DBH = diameter at breast height; H = stem height; V = stem volume; Lignin = lignin content; Holocellulose = holocellulose content; α-cellulose = α-cellulose content; Hemicellulose = hemicellulose content.

The Ref allele and Alt allele strings include the base before the InDel.
