# Supplementary material for: Dissection of Insertion–Deletion Variants within Differentially Expressed Genes Involved in Wood Formation in Populus
Source: Front Plant Sci. 2018 Jan 18;8:2199. doi: 10.3389/fpls.2017.02199 (PMC5778123; doi:10.3389/fpls.2017.02199)
Supplement: Supplementary file 8 [file Table_4.doc]

**Table S4** Detailed information on the additive and dominance effects of all significant InDels for each trait in the *P. tomentosa* natural population at a threshold value of FDR *q* < 0.10

| **Trait** | **Marker** | **Gene model** | **Locus** | **Mutation** | | **Additive model** | | **Dominance model** | |
| --- | --- | --- | --- | --- | --- | --- | --- | --- | --- |
|  |  |  |  |  |  |  |  |  |  |
|  |  |  |  | **Ref allele** | **Alt alleles** | ***P*-value** | **Effect** | ***P*-value** | **Effect** |
| α-cellulose | Potri.002G114200_02 | Potri.002G114200 | 3'UTR-downstream | CATG | C | 8.18E-03 | 4.84 | 6.90E-03 | 5.81 |
| α-cellulose | Potri.004G228800_03 | Potri.004G228800 | intron | AGAC | A | / | / | 2.53E-03 | 7.44 |
| α-cellulose | Potri.006G257100_02 | Potri.006G257100 | 3'UTR-downstream | A | AAAAATCT | 8.31E-03 | 3.17 | / | / |
| α-cellulose | Potri.008G161200_05 | Potri.008G161200 | intron | ATATATATAT | A | 8.80E-05 | 8.46 | 2.79E-04 | 9.79 |
| α-cellulose | Potri.012G037900_02 | Potri.012G037900 | 3'UTR-downstream | G | GTTTAAGTTTTAAGTCTTAAGT | 2.92E-03 | 4.54 | / | / |
| α-cellulose | Potri.012G044600_02 | Potri.012G044600 | exon | T | TGG | 9.54E-03 | 3.55 | / | / |
| α-cellulose | Potri.013G068900_01 | Potri.013G068900 | 3'UTR-downstream | TTTC | T | / | / | 5.84E-04 | -10.74 |
| α-cellulose | Potri.014G106600_02 | Potri.014G106600 | 3'UTR-downstream | T | TATTA | 1.52E-03 | 5.31 | / | / |
| α-cellulose | Potri.014G123000_02 | Potri.014G123000 | intron | A | ATAT | / | / | 9.09E-03 | 6.10 |
| α-cellulose | Potri.018G028300_01 | Potri.018G028300 | promoter | TTCA | T | / | / | 2.35E-03 | 7.12 |
| MFA | Potri.003G058600_01 | Potri.003G058600 | promoter | AGACATGCATATG | A | / | / | 9.13E-03 | -2.50 |
| MFA | Potri.008G112200_01 | Potri.008G112200 | promoter | AGC | A | 6.39E-04 | 3.14 | 6.71E-04 | -3.65 |
| MFA | Potri.014G018200_01 | Potri.014G018200 | promoter | C | CCTATG | / | / | 4.16E-04 | -3.42 |
| DBH | Potri.001G199100_01 | Potri.001G199100 | 3'UTR-downstream | A | AGT | / | / | 4.10E-03 | 4.40 |
| DBH | Potri.001G226100_01 | Potri.001G226100 | promoter | C | CCA | 1.51E-03 | 3.10 | / | / |
| DBH | Potri.001G266400_01 | Potri.001G266400 | intron within 5'UTR | T | TTTTTTTTTATTTTAGA | 1.36E-03 | 2.59 | / | / |
| DBH | Potri.002G197000_01 | Potri.002G197000 | promoter | G | GAGTATTCA | 2.12E-03 | 2.15 | / | / |
| DBH | Potri.004G051600_01 | Potri.004G051600 | 3'UTR-downstream | GCCTCC | G | 3.36E-04 | 3.33 | / | / |
| DBH | Potri.006G024300_04 | Potri.006G024300 | 3'UTR | AAG | A | 1.60E-04 | 2.80 | / | / |
| DBH | Potri.006G127500_01 | Potri.006G127500 | 3'UTR-downstream | A | AGGATTAT | 8.34E-05 | 3.15 | / | / |
| DBH | Potri.006G251300_04 | Potri.006G251300 | promoter | G | GAT | 8.17E-04 | 3.43 | / | / |
| DBH | Potri.007G016400_01 | Potri.007G016400 | intron | AAC | A | 1.63E-03 | 5.08 | / | / |
| DBH | Potri.008G082100_01 | Potri.008G082100 | 3'UTR | TGCG | T | 1.28E-03 | 4.28 | / | / |
| DBH | Potri.008G094700_02 | Potri.008G094700 | 3'UTR | A | AAG | 2.98E-03 | 3.39 | / | / |
| DBH | Potri.008G094700_05 | Potri.008G094700 | 5'UTR | GACAATCA | G | 4.94E-03 | 3.33 | / | / |
| DBH | Potri.008G097600_04 | Potri.008G097600 | intron | C | CTA | 1.93E-03 | 2.43 | / | / |
| DBH | Potri.008G116500_01 | Potri.008G116500 | 3'UTR-downstream | C | CCT | / | / | 4.20E-04 | -4.62 |
| DBH | Potri.012G040600_01 | Potri.012G040600 | promoter | TTC | T | 8.41E-04 | 3.36 | / | / |
| DBH | Potri.013G066000_02 | Potri.013G066000 | promoter | TTC | T | 7.03E-04 | 3.69 | / | / |
| DBH | Potri.013G067000_01 | Potri.013G067000 | 3'UTR-downstream | CTTTTTA | C | 8.60E-04 | 3.30 | / | / |
| DBH | Potri.013G154700_01 | Potri.013G154700 | intron | A | ACT | 1.14E-03 | 2.71 | / | / |
| DBH | Potri.014G017700_01 | Potri.014G017700 | 3'UTR | A | ACT | 2.71E-03 | 3.97 | / | / |
| DBH | Potri.014G106600_01 | Potri.014G106600 | promoter | GTAA | G | 3.26E-03 | 2.15 | / | / |
| DBH | Potri.014G106600_02 | Potri.014G106600 | 3'UTR-downstream | T | TATTA | 1.29E-03 | 3.73 | / | / |
| DBH | Potri.014G120700_01 | Potri.014G120700 | 3'UTR | A | AAAG | 3.67E-04 | 3.91 | / | / |
| DBH | Potri.014G121000_01 | Potri.014G121000 | promoter | T | TAA | 6.26E-05 | 2.76 | 6.06E-03 | -3.28 |
| DBH | Potri.017G014400_02 | Potri.017G014400 | promoter | ACAGG | A | 2.96E-03 | 3.02 | / | / |
| DBH | Potri.018G028300_01 | Potri.018G028300 | promoter | TTCA | T | 7.67E-03 | 3.05 | / | / |
| DBH | Potri.018G145800_02 | Potri.018G145800 | intron | TTGA | T | 6.52E-05 | 3.52 | / | / |
| DBH | Potri.018G145800_03 | Potri.018G145800 | intron | A | AAT | 6.77E-05 | 3.51 | / | / |
| DBH | Potri.018G145800_04 | Potri.018G145800 | intron | C | CAATG | / | / | 3.69E-04 | -4.34 |
| Fibre length | Potri.001G372400_01 | Potri.001G372400 | intron | A | ATAT | / | / | 4.63E-04 | -0.13 |
| Fibre length | Potri.008G118300_01 | Potri.008G118300 | promoter | TTTTG | T | 5.94E-04 | 0.05 | / | / |
| Fibre length | Potri.016G013700_01 | Potri.016G013700 | promoter | CCATTTA | C | / | / | 5.91E-03 | 0.04 |
| Fibre width | Potri.001G055700_02 | Potri.001G055700 | promoter | A | AACCT | / | / | 5.99E-04 | 2.04 |
| Fibre width | Potri.003G099700_02 | Potri.003G099700 | 3'UTR-downstream | C | CAAACT | 5.19E-03 | 0.97 | 1.69E-03 | 1.60 |
| Fibre width | Potri.008G112200_01 | Potri.008G112200 | promoter | AGC | A | / | / | 7.17E-03 | 1.52 |
| Fibre width | Potri.010G099700_01 | Potri.010G099700 | 3'UTR-downstream | TTA | T | / | / | 1.15E-03 | -1.65 |
| Fibre width | Potri.011G148000_01 | Potri.011G148000 | 3'UTR-downstream | G | GGTAC | 5.60E-03 | 1.38 | / | / |
| Fibre width | Potri.011G148200_01 | Potri.011G148200 | intron | T | TGGATATC | / | / | 1.83E-04 | 3.40 |
| Fibre width | Potri.012G037300_01 | Potri.012G037300 | 3'UTR-downstream | CTAAT | C | 1.45E-05 | 1.98 | 2.60E-03 | -2.01 |
| Fibre width | Potri.013G154700_01 | Potri.013G154700 | intron | A | ACT | / | / | 7.20E-03 | 1.22 |
| Fibre width | Potri.014G022400_01 | Potri.014G022400 | promoter | G | GCTGTGGTC | / | / | 2.39E-03 | 2.01 |
| Fibre width | Potri.014G121900_01 | Potri.014G121900 | intron | C | CTTG | / | / | 9.07E-03 | 1.87 |
| H | Potri.003G142300_02 | Potri.003G142300 | intron | T | TTA | 1.31E-03 | 1.12 | / | / |
| H | Potri.006G270700_06 | Potri.006G270700 | intron | TTTATG | T | 1.72E-03 | 1.81 | / | / |
| H | Potri.008G080800_06 | Potri.008G080800 | 3'UTR | TTTTCGCCCTCATCTAGTC | T | 5.39E-03 | 1.39 | / | / |
| H | Potri.008G161200_05 | Potri.008G161200 | intron | ATATATATAT | A | 9.15E-04 | 1.94 | 4.21E-03 | -2.16 |
| H | Potri.014G016300_01 | Potri.014G016300 | 5'UTR | T | TCC | / | / | 1.71E-03 | -1.80 |
| Hemicellulose | Potri.001G453600_01 | Potri.001G453600 | promoter | G | GCTT | 8.33E-04 | 5.46 | / | / |
| Hemicellulose | Potri.007G076500_03 | Potri.007G076500 | 3'UTR-downstream | C | CCGG | 5.07E-04 | 5.52 | / | / |
| Hemicellulose | Potri.008G089900_02 | Potri.008G089900 | intron | G | GTT | 1.65E-03 | 3.66 | / | / |
| Hemicellulose | Potri.009G123600_01 | Potri.009G123600 | promoter | A | AAAAT | 1.22E-03 | 5.51 | / | / |
| Hemicellulose | Potri.012G037900_02 | Potri.012G037900 | 3'UTR-downstream | G | GTTTAAGTTTTAAGTCTTAAGT | 3.98E-03 | 4.28 | / | / |
| Hemicellulose | Potri.015G073800_02 | Potri.015G073800 | intron | TAAA | T | 2.79E-03 | 4.77 | 8.95E-04 | 8.53 |
| Hemicellulose | Potri.016G068200_02 | Potri.016G068200 | intron | CAAGTAGTG | C | / | / | 1.99E-03 | -6.86 |
| Hemicellulose | Potri.016G090300_02 | Potri.016G090300 | 3'UTR | GGAAAA | G | / | / | 4.85E-03 | 6.56 |
| Holocellulose | Potri.001G453600_03 | Potri.001G453600 | promoter | A | AGC | 1.38E-03 | 5.08 | / | / |
| Holocellulose | Potri.010G100200_05 | Potri.010G100200 | promoter | AAAAAAAAAG | A | 5.63E-03 | 4.09 | / | / |
| Holocellulose | Potri.016G066300_01 | Potri.016G066300 | promoter | CGTCAAAACAGGTTAACCT | C | 9.73E-04 | 4.84 | / | / |
| Lignin | Potri.006G251300_02 | Potri.006G251300 | promoter | CAA | C | / | / | 5.41E-04 | 2.07 |
| Lignin | Potri.008G082100_01 | Potri.008G082100 | 3'UTR | TGCG | T | 5.83E-05 | 2.18 | / | / |
| Lignin | Potri.009G095800_01 | Potri.009G095800 | intron | T | TTAC | / | / | 7.04E-04 | 2.30 |
| Lignin | Potri.011G059300_02 | Potri.011G059300 | promoter | T | TAG | / | / | 9.10E-05 | 2.47 |
| Lignin | Potri.013G067500_05 | Potri.013G067500 | 3'UTR-downstream | GTTTTTTGTTTTTTC | G | 7.46E-04 | 1.07 | / | / |
| Lignin | Potri.013G070200_03 | Potri.013G070200 | 3'UTR-downstream | T | TAA | / | / | 4.73E-03 | 1.70 |
| Lignin | Potri.013G078500_02 | Potri.013G078500 | intron | TATCCCAGTACCCCC | T | 2.19E-03 | 1.86 | / | / |
| Lignin | Potri.018G098900_01 | Potri.018G098900 | intron | T | TATAAAC | 1.67E-03 | 1.53 | / | / |
| V | Potri.001G266400_01 | Potri.001G266400 | intron within 5'UTR | T | TTTTTTTTTATTTTAGA | 2.46E-03 | 0.18 | / | / |
| V | Potri.004G051600_01 | Potri.004G051600 | 3'UTR-downstream | GCCTCC | G | 1.83E-03 | 0.20 | / | / |
| V | Potri.006G024300_04 | Potri.006G024300 | 3'UTR | AAG | A | 5.76E-04 | 0.19 | / | / |
| V | Potri.006G127500_01 | Potri.006G127500 | 3'UTR-downstream | A | AGGATTAT | 7.91E-05 | 0.21 | / | / |
| V | Potri.006G251300_04 | Potri.006G251300 | promoter | G | GAT | 6.20E-03 | 0.19 | / | / |
| V | Potri.008G094000_05 | Potri.008G094000 | 3'UTR-downstream | GGGGA | G | 1.93E-03 | 0.30 | / | / |
| V | Potri.008G094700_02 | Potri.008G094700 | 3'UTR | A | AAG | 2.29E-03 | 0.23 | / | / |
| V | Potri.008G116500_01 | Potri.008G116500 | 3'UTR-downstream | C | CCT | / | / | 2.00E-03 | -0.28 |
| V | Potri.008G161200_05 | Potri.008G161200 | intron | ATATATATAT | A | 1.07E-04 | 0.34 | / | / |
| V | Potri.012G040600_01 | Potri.012G040600 | promoter | TTC | T | 3.79E-05 | 0.25 | 2.84E-03 | -0.25 |
| V | Potri.013G064700_02 | Potri.013G064700 | intron | TTC | T | 6.15E-04 | 0.22 | / | / |
| V | Potri.013G066000_02 | Potri.013G066000 | promoter | TTC | T | 4.73E-03 | 0.21 | / | / |
| V | Potri.013G067000_01 | Potri.013G067000 | 3'UTR-downstream | CTTTTTA | C | 2.96E-03 | 0.20 | / | / |
| V | Potri.013G068700_01 | Potri.013G068700 | intron | T | TAAAAGAAAAG | 6.35E-03 | 0.19 | / | / |
| V | Potri.014G017700_01 | Potri.014G017700 | 3'UTR | A | ACT | 3.88E-04 | 0.33 | / | / |
| V | Potri.014G106600_01 | Potri.014G106600 | promoter | GTAA | G | 5.30E-03 | 0.15 | / | / |
| V | Potri.014G106600_02 | Potri.014G106600 | 3'UTR-downstream | T | TATTA | 5.35E-03 | 0.22 | / | / |
| V | Potri.014G121000_01 | Potri.014G121000 | promoter | T | TAA | 4.69E-04 | 0.16 | / | / |
| V | Potri.018G145800_02 | Potri.018G145800 | intron | TTGA | T | 2.10E-04 | 0.21 | / | / |
| V | Potri.018G145800_03 | Potri.018G145800 | intron | A | AAT | 2.56E-04 | 0.21 | / | / |
| V | Potri.018G145800_04 | Potri.018G145800 | intron | C | CAATG | / | / | 1.30E-03 | -0.26 |

*P*-value = significant level for association (FDR *q* < 0.10).

MFA = microfiber angle; DBH = diameter at breast height; H = stem height; V = stem volume; Lignin = lignin content; Holocellulose = holocellulose content; α-cellulose = α-cellulose content; Hemicellulose = hemicellulose content.

The Ref allele and Alt allele strings include the base before the InDel.
