# Supplementary material for: Dissection of Insertion–Deletion Variants within Differentially Expressed Genes Involved in Wood Formation in Populus
Source: Front Plant Sci. 2018 Jan 18;8:2199. doi: 10.3389/fpls.2017.02199 (PMC5778123; doi:10.3389/fpls.2017.02199)
Supplement: Supplementary file 9 [file Table_5.DOC]

**Table S5** Summary of 7 significant differentially expressed genes among three genotypic classes at *P* < 0.01

| **Maker** | **Gene model** | **Location** | **Ref allele** | **Alt alleles** |
| --- | --- | --- | --- | --- |
| Potri.001G055700_02 | Potri.001G055700 | promoter | A | AACCT |
| Potri.008G161200_05 | Potri.008G161200 | intron3 | ATATATATAT | A |
| Potri.009G095800_01 | Potri.009G095800 | intron2 | T | TTAC |
| Potri.011G059300_02 | Potri.011G059300 | promoter | T | TAG |
| Potri.012G044600_02 | Potri.012G044600 | exon2 | T | TGG |
| Potri.014G106600_02 | Potri.014G106600 | 3'UTR-downstream | T | TATTA |
| Potri.016G090300_02 | Potri.016G090300 | 3'UTR | GGAAAA | G |

The Ref allele and Alt allele strings include the base before the InDel.
