# Supplementary material for: Dissection of Insertion–Deletion Variants within Differentially Expressed Genes Involved in Wood Formation in Populus
Source: Front Plant Sci. 2018 Jan 18;8:2199. doi: 10.3389/fpls.2017.02199 (PMC5778123; doi:10.3389/fpls.2017.02199)
Supplement: Supplementary file 10 [file Table_6.DOC]

**Table S6** Two epistatic gene-gene interactions underlying the monolignol biosynthesis pathway with A × A epistatic effects between four lignin-related genes

| **Locus1** | **Location1** | **Gene model 1** | **Ref. gene**  **name 1** | **Main effect** | **Ref/Alt allele** | **Locus2** | **Location2** | **Gene model 2** | **Ref. gene name 2** | **Main effect** | **Ref/Alt allele** | **Trait** | | **Type** | **Epistasis effect** | ***P*_value** |
| --- | --- | --- | --- | --- | --- | --- | --- | --- | --- | --- | --- | --- | --- | --- | --- | --- |
| Potri.002G183600_01 | intron | Potri.002G183600 | *PtrCCoAOMT6* | \ | A/ACAGTACAGCAGCCAG | Potri.005G248500_02 | 3'UTR-downstream | Potri.005G248500 | *Ptr4CL7* | / | TAA/T | | Lignin | AA | -2.65 | 1.18E-05 |
| Potri.010G104400_02 | intron | Potri.010G104400 | *PtrCCoAOMT4* | \ | A/ATTTGT | Potri.003G099700_04 | 3'UTR-downstream | Potri.003G099700 | *Ptr4CL9* | \ | A/AAAT | | Lignin | AA | 3.57 | 3.09E-05 |

*P*-value = significant level for association; Main effect = additive (a) or dominance (d) effect.

Lignin = lignin content.

AA = additive × additive epistasis effects.
