# Supplementary material for: Dissection of Insertion–Deletion Variants within Differentially Expressed Genes Involved in Wood Formation in Populus
Source: Front Plant Sci. 2018 Jan 18;8:2199. doi: 10.3389/fpls.2017.02199 (PMC5778123; doi:10.3389/fpls.2017.02199)
Supplement: Supplementary file 12 [file Methods_S1.DOC]

**Method S1**

1. **Population materials preparation for resequencing**

In the 1980s, a total of 1,047 native *Populus tomentosa* individuals were collected across the entire natural distribution range of *P. tomentosa*, extending over an area of 1 million km^2^ (30°N-40°N, 105°E-125°E). Root segments were then used to establish a clonal arboretum by a randomized complete block design with three replications in Guan Xian County, Shandong Province, China (36°23’N, 115°47’E). The association population consisting of 435 24-year-old, unrelated natural *Populus tomentosa* individuals was randomly sampled from this clonal arboretum with three replications in 2013. The distribution zone of *P*. *tomentosa* was divided into three large climatic regions, Northeastern, Northwestern, and Southern, based on a principal components analysis and isolate fuzzy clustering using 16 meteorological factors (Huang, 1992). Du *et al*. (2012) further estimated the population structure and resulted in three subpopulations which were consistent well with the three climatic regions. Fresh leaves were collected and total genomic DNA was extracted using the DNeasy Plant Mini kit (Qiagen, Shanghai, China) following the manufacturer’s protocol. The methods about generating phenotypic data and the Pearson’s correlation tests of these traits were detailed described by Du *et al.* (2014), and results showed that the correlated traits used in this study tended to be biologically related and shared the genetic bases.

1. **Material preparation for transcriptome sequencing and RT-PCR**

Three-year-old *Populus tomentosa* (clone “LM50”) were planted in the national nursery in Guan Xian County, Shandong Province, China (36°23’N, 115°47’E). For biological replicates, this study used three individual, three-year-old clones from one genotype of *P. tomentosa*. For RNA extraction, fresh tissue samples of leaf and apex were collected from the 1-year-old vegetative propagated cuttings. According to Li*et al.* (2009), the developing xylem tissues were collected by scraping the thin (approximately 1.0 mm) and partially lignified layer on the exposed xylem surface at breast height. Mature xylem tissues were collected by scraping the deep layer on the exposed xylem surface at breast height (Zhang et al*.* 2010). The other wood forming tissues, including phloem and cambium, were collected as described (Djerbi et al*.* 2004).

1. **Paired-end cDNA library construction**

Total RNA extraction was performed by a modified CTAB method in which isopropanol was used for RNA precipitation instead of lithium chloride (Chen et al. 2015). Additional purifying of total RNA was performed using the RNeasy micro kit (Cat#74004, Qiagen). Then, the total RNA was used for the paired-end cDNA library construction following the TruSeq RNA Sample Preparation Guide (Illumina). After purifying the poly-A containing mRNA by poly-T oligo-attached magnetic beads, the mRNA was fragmented with divalent cations and heat. Then the cleaved RNA fragments were reverse transcribed into first strand cDNA using reverse transcriptase and random primers, followed by second strand cDNA synthesis using DNA polymerase I and RNaseH. This double-stranded cDNA was subjected to end-repair, followed by 3'-adenylated using Klenow exo-polymerase. Mutiple indexing adapters were ligated to the ends of these 3'-adenylated cDNA fragments. Adaptor-ligated fragments were separated by size on an agarose gel, and the desired range of cDNA fragments (200 ± 25 bp) were excised from the gel. Then PCR was used to selectively enrich and amplify the cDNA fragments. After validation on an Agilent Technologies 2100 Bioanalyzer using the Agilent DNA 1000 chip kit, the cDNA library was sequenced on Illumina HiSeq2000 sequencing platform and 100 bp paired-end reads (sequencing depth~30X, 15G) were generated at Shanghai Bio Institute.

1. **Transcriptome analysis process**

Quality control of raw reads was carried out by FastX software (v0.0.13, <http://hannonlab.cshl.edu/fastx_toolkit/>) as the following process, including: 1) removing the reads with low overall quality; 2) removing the reads with the proportion of the bases ≤ 50% but Q > 20 (Q = -10^logerror_ratio^); 3) discarding the 3' end bases with Q < 10; 4) discarding the adapter sequences; 5) removing the reads ≥ 10% with ambiguous bases ‘N’; 6) removing the reads shorter than 20 nucleotides; 7) removing the ribosome RNA reads. The qualified reads which passed the filters above were aligned to the *Populus trichocarpa* reference genome v3.0 (ftp://plantgenie.org/Data/PopGenIE/Populus_trichocarpa/v3.0/) using the splice mapping algorithm integrated in TopHat (v2.0.9, <http://tophat.cbcb.umd.edu/>) with default parameters, excepting for the multiple hits with *-g 1*.

1. **Differentially expressed gene analysis**

Cufflinks (version 2.1.1) was used to calculate the expression level of each gene with *Populus trichocarpa* reference genome v3.0 as GFF format, and reported it by “fragments per kilobase of transcript per million fragments mapped” or FPKM (Trapnell et al. 2010). FPKM was defined as follow:


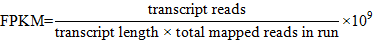


Here, we did not consider the alternative splicing and “transcript reads” referred to the reads covering all exon(s) of a gene. The fold change (FC) was performed between each two tissues using FPKM. To identify significant DE genes, Cuffdiff program in Cufflinks v2.1.1 (Trapnell et al. 2010) was used to perform differential expression. Then the false discovery rate control was performed using *p*-value, genes with the fold change (FC) ≥ 2 or ≤ 0.5 and *q* value of 0.10 with a *p* value of 1.0e-03 were selected as differentially expressed genes.

1. **Gene ontology (GO) analysis**

GO terms were performed by AgriGO (<http://bioinfo.cau.edu.cn/agriGO/index.php>) and the enriched GO terms were checked with a false discovery rate (FDR) value of 0.05 as the cut-off for significant GO terms.

1. **Feasibility analysis of using *Populus trichocarpa* genome as a reference for mapping and InDel calling of *Populus tomentosa***

We estimated the divergence of the genic / non-genic space between *P. trichocarpa* and *P. tomentosa*. The resequencing reads from four *P. tomentosa* individuals were mapped to the *P. trichocarpa* using BWA-mem with default parameters (v0.7.13-r1126; H. Li & Durbin, 2009). The mapping rates are ranging from 89.58% to 89.98%. Secondly, 40 randomly selected pacbio long reads (average length 3,990 bp) generated from the sequencing of *P. tomentosa* were aligned to the *P. trichocarpa* genome using blat software with default parameters. A total of 15 and 16 sequences were mapped to the genic regions and non-genic regions of *P. trichocarpa* sequence with significant hits (coverage above 0.8), and identity of genic / non-genic space were calculated according to the Blast definition (Identities / Columns). The average identity was 97.2% and 97.6% in non-genic and genic regions, respectively, suggesting it is feasible to use the *P. trichocarpa* genome sequence as a template for *P. tomentosa* for mapping and InDel calling.

1. **Resequencing of the association population and InDel calling**

All 435 unrelated individuals were sequenced with an average of 15X coverage (raw data) using the Illumina GA2 platform and the quality control of paired-end short reads of 100 bp was performed by removing the low-quality reads containing contiguous undetermined nucleotides (≥ 50% of nucleotides with a quality score < Q20. Then the paired-end short reads were mapped and aligned to the *P. trichocarpa* reference genome v3.0 using SOAPaligner/SOAP2 v2.20 with the default options (R. Li et al. 2008). As a consequence, the mapping rate in different accessions varied from 81% to 92%, and the effective mapping depth was ~11X for most individuals. To get high-quality InDels, only the uniquely mapped paired-end reads were used for small InDels calling. InDel calling and genotype calling was performed using the GATK v3 (<https://www.broadinstitute.org/gatk/>) with default parameters. Specially, a process named IndelRealigner in GATK was performed to sensitively and specifically identify InDels. Then this original InDel data was filtered by Variant Call Format (VCF) tool v4.1 (Danecek et al. 2011) with missing rate ≤ 0.25 and MAF > 0.001.

According to the positions of differentially expressed genes on *P. trichocarpa* reference genome v3.0, InDels for the DE genes were extracted by a custom Python script. We also extracted InDels within 2,000 bp sequences upstream of the first start codon (promoter) and 500 bp sequences downstream of the stop codon (3'UTR). In order to get clean InDel genotypes for association, we further filtered the genotype data as following process: 1) removing the complex InDels sites and the diallelic InDels sites with minor allele frequency < 0.05; 2) removing the InDel sites showed same genotypes among 435 *P. tomentosa* individuals; 3) removing the InDel sites with the minor genotype frequency < 5%. In addition, we randomly validated several of the genic-InDels by comparing them with previous sequencing data of 30 genes in the same population. InDel sizes were confirmed by capillary electrophoresis on a capillary sequencer ABI3730xl DNA Analyzer (Applied Biosystems, Carlsbad, CA, USA) and found that the accuracy of genic-InDel calling reached 96.7% (Supplementary Data S1).

Some researches showed that the genomes of *Populus euphratica* and *Salix suchowensis* both exhibited extensive collinearity with *P. trichocarpa* (Ma et al. 2013, Dai et al. 2014), suggesting that the genome of *P. tomentosa* should have a good collinearity with *P. trichocarpa*. Therefore, we used the *P. trichocarpa* genome as a reference to draw the circular diagram by Circos visualization tool.11 (Krzywinski et al. 2009). The locations of DE genes were based on the locations on the *P. trichocarpa* genome.

**Reference**

1. Chen J, Chen B, Zhang D (2015) Transcript profiling of *Populus tomentosa* genes in normal, tension, and opposite wood by RNA-seq. BMC Genomics 16: 164.
2. Dai X, Hu Q, Cai Q et al. (2014) The willow genome and divergent evolution from poplar after the common genome duplication. Cell Res 24: 1274.
3. Danecek P, Auton A, Abecasis G et al. (2011) The variant call format and VCFtools. Bioinformatics 27: 2156-2158.
4. Djerbi S, Aspeborg H, Nilsson P et al. (2004) Identification and expression analysis of genes encoding putative cellulose synthases (*CesA*) in the hybrid aspen, *Populus tremula* (L.) × *P. tremuloides* (Michx.). Cellulose 11: 301-312.
5. Du Q, Wang B, Wei Z, Zhang D, Li B (2012) Genetic diversity and population structure of Chinese white poplar (*Populus tomentosa*) revealed by SSR markers. J Hered 103: 853-862.
6. Du Q, Xu B, Gong C et al. (2014) Variation in growth, leaf, and wood property traits of Chinese white poplar *Populus tomentosa*, a major industrial tree species in Northern China. Can J Forest Res 44: 326-339.
7. Huang, Z.H. **(**1992) The study on the climatic regionalization of the distributional region of *Populus tomentosa*. Journal of Beijing Forestry University 14: 26-32.
8. Krzywinski M, Schein J, Birol I et al. (2009) Circos: an information aesthetic for comparative genomics. Genome Res 19: 1639-1645.
9. Li H, Durbin R (2009) Fast and accurate short read alignment with Burrows-Wheeler transform. Bioinformatics 25: 1754-1760.
10. Li R, Li Y, Kristiansen K, Wang J (2008) SOAP: short oligonucleotide alignment program. Bioinformatics 24: 713-714.
11. Li X, Wu HX, Dillon SK, Southerton SG (2009) Generation and analysis of expressed sequence tags from six developing xylem libraries in *Pinus radiata* D. Don. BMC Genomics 10: 1.
12. Ma T, Wang J, Zhou G et al. (2013) Genomic insights into salt adaptation in a desert poplar. Nat Commun 4.
13. Trapnell C, Williams BA, Pertea G et al. (2010) Transcript assembly and quantification by RNA-Seq reveals unannotated transcripts and isoform switching during cell differentiation. Nat Biotechnol 28: 511-515.
14. Zhang D, Du Q, Xu B, Zhang Z, Li B. (2010) The actin multigene family in *Populus*: organization, expression and phylogenetic analysis. Mol Genet Genomics 284: 105-119.
